# Disturbance of floral colour pattern by activation of an endogenous pararetrovirus, petunia vein clearing virus, in aged petunia plants

**DOI:** 10.1111/tpj.14728

**Published:** 2020-04-03

**Authors:** Kazunori Kuriyama, Midori Tabara, Hiromitsu Moriyama, Akira Kanazawa, Hisashi Koiwa, Hideki Takahashi, Toshiyuki Fukuhara

**Affiliations:** ^1^ Department of Applied Biological Sciences Tokyo University of Agriculture and Technology 3‐5‐8 Saiwaicho Fuchu Tokyo 183‐8509 Japan; ^2^ Institute of Global Innovation Research Tokyo University of Agriculture and Technology 3‐5‐8 Saiwaicho Fuchu Tokyo 183‐8509 Japan; ^3^ Research Faculty of Agriculture Hokkaido University Kita 9, Nishi 9, Kita‐ku Sapporo 060‐8589 Japan; ^4^ Department of Horticultural Sciences Texas A&M University College Station TX 77843 USA; ^5^ Graduate School of Agricultural Science Tohoku University 468‐1, Aramaki‐Aza‐Aoba Sendai 980‐0845 Japan

**Keywords:** petunia vein clearing virus, petunia, bicolour petal, post‐transcriptional gene silencing, DNA methylation, pararetrovirus, ageing

## Abstract

White areas of star‐type bicolour petals of petunia (*Petunia hybrida*) are caused by post‐transcriptional gene silencing (PTGS) of the key enzyme of anthocyanin biosynthesis. We observed blotched flowers and a vein‐clearing symptom in aged petunia plants. To determine the cause of blotched flowers, we focused on an endogenous pararetrovirus, petunia vein clearing virus (PVCV), because this virus may have a suppressor of PTGS (VSR). Transcripts and episomal DNAs derived from proviral PVCVs accumulated in aged plants, indicating that PVCV was activated as the host plant aged. Furthermore, DNA methylation of CG and CHG sites in the promoter region of proviral PVCV decreased in aged plants, suggesting that poor maintenance of DNA methylation activates PVCV. In parallel, *de novo* DNA methylation of CHH sites in its promoter region was also detected. Therefore, both activation and inactivation of PVCV occurred in aged plants. The accumulation of PVCV transcripts and episomal DNAs in blotched regions and the detection of VSR activity support a mechanism in which suppression of PTGS by PVCV causes blotched flowers.

## Significance Statement

The activation process of an endogenous pararetrovirus, petunia vein clearing virus (PVCV), and the effect of its activation on the bicolour pattern of star‐type petunia were observed. During the long‐term cultivation of petunia plants, the methylation level of the promoter region of PVCV proviruses decreased, an activated PVCV interfered with post‐transcriptional chalcone synthase gene silencing by its suppressor of RNA silencing, and then blotched flowers appeared.

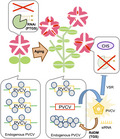

## INTRODUCTION

Various transposable elements make up a large fraction of the eukaryotic genome and contribute to genomic diversity. These transposable elements (transposons) are primarily classified into two types, DNA transposons, and retrotransposons. A retrotransposon is transcribed from DNA to RNA, and the RNA transcript is then reverse transcribed to DNA by its own reverse transcriptase. This copied DNA is then inserted into the genome’s DNA at a new position (Feschotte *et al.*, 2002; Lisch, 2013). Retrotransposons are closely related to retroviruses and pararetroviruses. Various endogenous retrovirus‐ and pararetrovirus‐related sequences are found in almost all eukaryotic genomes.

Endogenous pararetrovirus sequences are the most abundant integrated viral sequences in plant genomes (Chabannes and Iskra‐Caruana, 2013). The sequences of most endogenous pararetroviruses can no longer propagate in the host, that is they are relics of virus endogenization, but some retain their proviral status with the ability to propagate and symptomatically affect their host plants. For instance, tobacco vein clearing virus (TVCV), banana streak virus (BSV), and petunia vein clearing virus (PVCV) originate from endogenous pararetroviruses in plants (Jakowitsch *et al.*, 1999; Ndowora *et al.*, 1999; Richet‐Pöggeler *et al.*, 2003). A functional pararetroviral sequence has one quasilong terminal repeat (QTR) sequence, which is similar to the long terminal repeat (LTR) sequence of retroviruses and contains a promoter sequence, and a signal sequence for adding a poly A to transcripts (Richet‐Pöggeler *et al.*, 2003; Noreen *et al.*, 2007). The life cycle of pararetroviruses is similar to that of retroviruses in which viral RNA is transcribed from the pararetroviral DNA (provirus) in its host genomic DNA. Then episomal DNA is generated from viral RNA transcript as a template by its own reverse transcriptase (Staginnus and Richert‐Pöggeler, 2006). This circularized episomal DNA, as a viral genome, is coated with capsid proteins to form a virus particle, which can move from cell to cell and from plant to plant.

PVCV is a member of the genus *Petuvirus* in the family *Caulimoviridae*. Cauliflower mosaic virus (CaMV) is a type species in the genus *Caulimovirus* in the family *Caulimoviridae* (Geering and Hull, 2012)*.* PVCV has a single molecule of non‐covalently closed circular double‐stranded DNA of 7.2 kbp, which encodes a single open reading frame (ORF) with significant similarity to ORFs encoded by other family members such as CaMV. This long ORF likely encodes a polyprotein whose several domains are cleaved into individual functional proteins by their own proteinase(s), using a similar mechanism as retrovirus polyproteins. However, the cleavage sites of putative PVCV polyprotein and the functions of proteins encoded by PVCV remain unknown. It has been reported that PVCV is activated under stress conditions such as heat, drought, and wounding (Richet‐Pöggeler *et al.*, 2003; Noreen *et al.*, 2007). The activation of PVCV causes disease symptoms to host plants such as vein clearing and yellowing and curling of leaves, in which episomal DNAs and virus particles are detectable (Richert‐Pöggeler *et al.*, 2003). Transmission of PVCV by mechanical infection has never been proven, but graft transmission between tobacco and petunia plants has been reported (Richert‐Pöggeler *et al.*, 2003). All cultivated petunia plants (*Petunia hybrida*) have many (*c.* 50 copies) PVCV proviruses within their genome’s DNA, which could be attributed to the presence of PVCV in a wild petunia species (*P. axillaris*) (Bombarely *et al.*, 2016). Consequently, all cultivated petunia plants potentially exhibit these disease symptoms.

Host plant cells control nucleic acid parasites such as transposons and viruses by RNA silencing (interference), a mechanism that transcriptionally (transcriptional gene silencing, TGS) or post‐transcriptionally (post‐transcriptional gene silencing, PTGS) downregulates gene expression of transposons and viruses to suppress their mobilization and propagation (Castel and Martienssen, 2013). In *Arabidopsis thaliana*, two endoribonucleases (Dicers), DCL3 and DCL4, cleave double‐stranded RNAs (dsRNAs) into 24‐nt and 21‐nt small interfering RNAs (siRNAs), respectively (Fukudome and Fukuhara, 2017). The products of DCL3, 24‐nt siRNAs, function in TGS via RNA‐directed DNA methylation (RdDM) to suppress the activation of transposons, presumably pararetroviruses (Matzke and Mosher, 2014). Conversely, the products of DCL4, 21‐nt siRNAs, function in PTGS (RNA interference, RNAi) via sequence‐specific RNA cleavage to regulate gene expression and defend against virus propagation (Garcia‐Ruiz *et al.*, 2010; Wang *et al.*, 2011).

**Figure 1:**
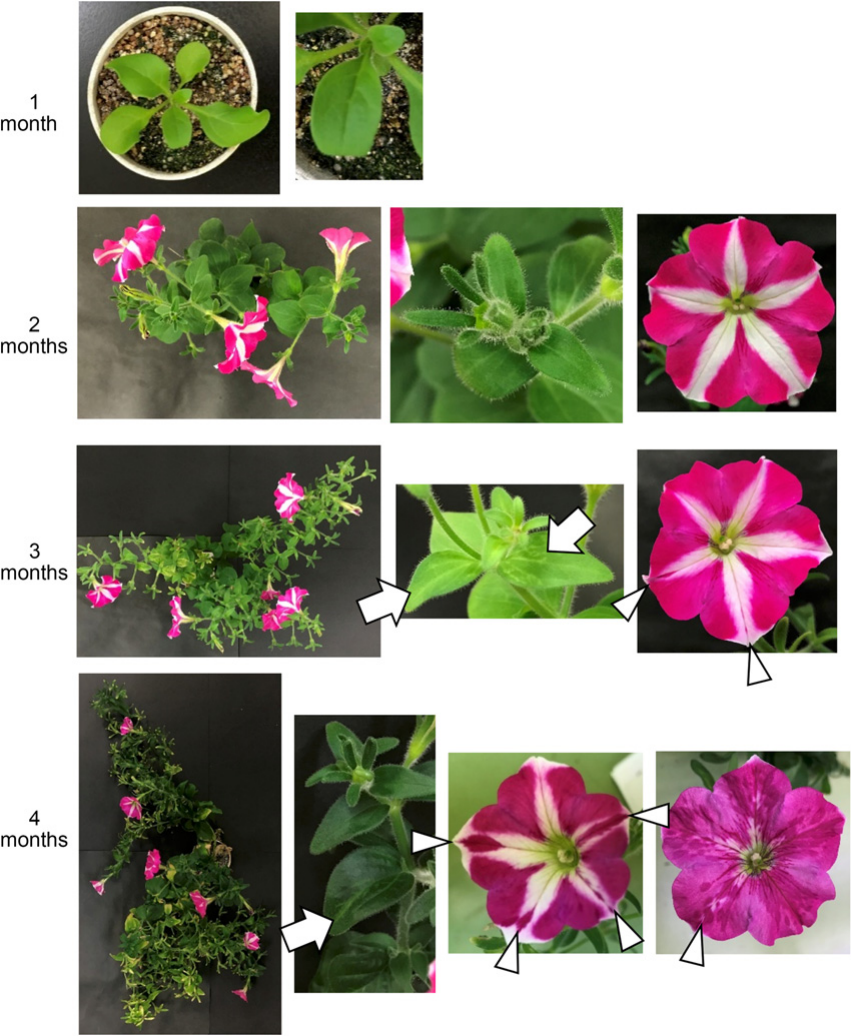
Activation of petunia vein clearing caulimovirus (PVCV) as a host plant ages. Photographs of plants, leaves, and flowers of petunia cv Rondo Rose‐Star are shown. Vein‐clearing symptoms and blotched flowers were observed in 3‐month‐old and 4‐month‐old plants. Blotches and vein‐clearing are indicated by white arrowheads and arrows, respectively. 1m, 2m, 3m, and 4m indicate 1‐, 2‐, 3‐, and 4‐month‐old petunia plants, respectively.

In addition to responding to invading nucleic acids, these mechanisms are involved in intrinsically suppressing endogenous genes and contribute to a particular plant phenotype. PTGS was first reported in transgenic plants and was referred to as co‐suppression, in which suppression of both a transgene and its homologous endogenous gene was induced by introduction of the transgene (Napoli *et al.*, 1990; van der Krol *et al.*, 1990). Suppression of endogenous genes through PTGS in the absence of a transgene was subsequently detected in non‐transgenic plants: most commonly, plants lack pigmentation in a particular tissue as a consequence of the suppression of the *CHS* gene for chalcone synthase, a key enzyme of anthocyanin biosynthesis. This phenomenon has been observed in the seed coat tissues of soybean (Senda *et al.*, 2004; Tuteja *et al.*, 2004), various tissues including maize kernels (Della Vedova *et al.*, 2005), and flower tissues of petunia (Koseki *et al.*, 2005) and dahlia (Ohno *et al.*, 2011). In a petunia variety ‘Red‐Star’, PTGS of the *CHS‐A* gene, a gene copy abundantly expressed in the flower tissues among the *CHS* gene family, is naturally induced in tissues along the mid‐vein of each petal, which results in the formation of a star‐type bicolour pattern (Koseki *et al.*, 2005) (for flower phenotype, see Figure 1). Similar naturally occurring PTGS is also implicated in the formation of non‐pigmented sectors in the outer edge of the petal tissues of a picotee‐type petunia variety (Morita *et al.*, 2012). A deep sequencing analysis uncovered commonality in siRNAs between naturally occurring PTGS and co‐suppression of the *CHS‐A* genes in petunia, indicating mechanistic similarities between these silencing systems (Kasai *et al.*, 2013). This finding thus provided a basis for the phenotypic resemblance between existing varieties and co‐suppressed plants of petunia, which was noticed by the authors of the above‐mentioned articles of co‐suppression published in the 1990s (Mittelsten Scheid, 2019).

Most plant viruses protect themselves from host RNA silencing (PTGS) by viral suppressors of RNA silencing (VSRs) (Burgyán and Havelda, 2011), and they also inhibit naturally occurring PTGS. Indeed, recovery of pigmentation in yellow seed coats of soybean by virus infection (Senda *et al.*, 2004) and in bicolour flowers of star‐type petunia by infection of cucumber mosaic virus (CMV), tobacco etch virus (TEV), and potato potyvirus Y (PVY) (Teycheney and Tepher, 2001; Koseki *et al.*, 2005; Griesbach *et al.*, 2007) has been observed. However, the relationship between host RNA silencing and endogenous pararetroviruses remains unknown.

Here we characterized the activation process of PVCV and the effect of its activation on the bicolour pattern of star‐type petunia. During the long‐term cultivation of host plants, the methylation levels of the promoter region of PVCV proviruses decreased, and PVCV transcription was activated. VSR of PVCV interferes with PTGS of the *CHS‐A* gene, restoring the expression of *CHS‐A* in flowers. Consequently, blotched flowers formed on aged plants. These results indicated that activation of a pararetrovirus during long‐term cultivation affects flower colour patterning in petunia.

## RESULTS

### Activation of PVCV as a host plant ages

**Figure 2:**
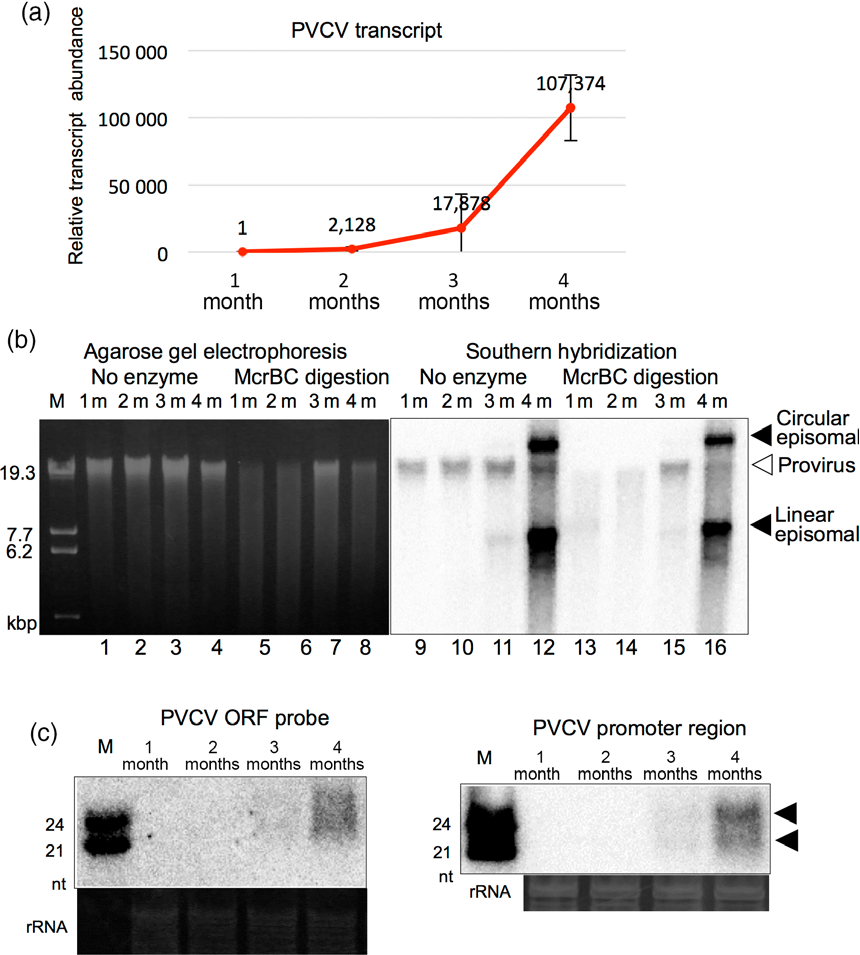
Activation of petunia vein clearing caulimovirus (PVCV) as the host plant ages. (a) Accumulation of the PVCV transcript as the plant aged. Relative abundance of PVCV transcripts in leaves of 1‐, 2‐, 3‐, and 4‐month‐old petunia plants (1m, 2m, 3m, and 4m, respectively) were determined by qPCR and normalized to the abundance of 26S rRNA. Total RNAs were prepared from several leaves of petunia plants. Error bars indicate the standard errors of three biological replications. (b) Detection of episomal PVCV DNAs and analysis of methylation states of petunia genomic DNA and proviral and episomal PVCV DNAs. Total DNAs prepared from several leaves and those digested by the methylation‐dependent restriction endonuclease McrBC were analyzed by agarose gel electrophoresis (left), and PVCV sequences were detected by Southern blot hybridization (right). Proviral PVCV sequences are indicated by a white arrowhead, and the linear form of episomal PVCV DNAs of *c.* 7.2 kbp and its putative circular form are indicated by black arrowheads. (c) siRNAs derived from the coding (left) and promoter (right) regions of PVCV were detected by northern blot hybridization. Total RNAs prepared from several leaves were used. siRNAs of 21‐nt and 24‐nt in length are indicated by black arrowheads. Ribosomal RNAs stained by ethidium bromide are shown as loading controls. Molecular weight markers of linear dsDNAs (b) and single‐stranded RNAs (ssRNAs) (c) are indicated as lane M.

It has been reported previously that a pararetorovirus, PVCV, is activated by environmental stresses such as heat, drought, and wounding (Zeidan *et al.*, 2000; Richert‐Pöggeler *et al.*, 2003). However, we did not detect any vein‐clearing symptoms in the star‐type petunia cultivar (cv) Rondo Rose‐Star by heat or drought stress treatment. Instead, we observed the vein‐clearing symptom in 3‐month‐old plants that were grown under normal growth conditions in a growth room (Figure 1). Numerous PVCV transcripts were detected from total RNAs prepared from petunia leaves with the vein‐clearing symptom using quantitative real‐time PCR (qPCR) analysis, and the level increased in an age‐dependent manner (Figure 2a). The vein‐clearing symptom was correlated with the accumulation of PVCV transcripts. Furthermore, the putative circular and linear forms of episomal DNAs containing the PVCV sequence were also detected in 3‐ and 4‐month‐old plants using Southern blot analysis (Figure 2b, lanes 11, 12, 15, and 16). Therefore, the appearance of the vein‐clearing symptom was associated with the accumulation of PVCV transcripts and episomal DNAs, which are the hallmarks of the activation of pararetroviruses. These results indicate that PVCV was activated as the host plants aged.

### Differences in nucleotide sequences between proviral and episomal DNAs of PVCV

The promoter regions of reported nucleotide sequences of proviral PVCV and its episomal DNA are slightly different (Richert‐Pöggeler *et al.*, 2003). We obtained nucleotide sequences of cDNAs derived from total RNAs prepared from 4‐month‐old petunia plants and compared them with proviral and episomal PVCV sequences deposited in the NCBI database (AY228106 and U95208.2; Richert‐Pöggeler *et al.*, 2003). Two types of PVCV transcripts were detected in 4‐month‐old plants of cv Rondo Rose‐Star (Figure S1). They contained polymorphisms (substitution, deletion, or insertion) at nine sites (Figure S1), which are similar to deposited nucleotide sequences of proviral and episomal PVCV sequences, respectively. In transcriptome (RNA‐seq) analysis using total RNAs prepared from 2‐ and 4‐month‐old plants, there were many more sequence reads derived from PVCV transcripts in 4‐month‐old plants than from 2‐month‐old plants (Table S1). In the RNA‐seq datasets obtained from 4‐month‐old plants, the majority of PVCV transcripts were derived from the episomal DNA in one plant (4m‐3 in Figure S2 and Table S2), but episomal transcripts were only a minority in another plant (4m‐2 in Figure S2 and Table S2). The ratio of PVCV reads per total reads in the dataset of a 4‐month‐old plant containing numerous episomal transcripts (4m‐3 in Table S1 and Figure S2) was more than that in another dataset containing a few episomal transcripts (4m‐2 in Table S1 and Figure S2), suggesting that viral transcripts increased enormously once the episomal DNA appeared.

### Age‐dependent alteration of DNA methylation level in proviral PVCV loci

We hypothesized that the methylation state of genomic DNA was associated with PVCV activation, and examined the methylation state of PVCV using the methylation‐dependent restriction endonuclease McrBC and Southern blot hybridization (Figure 2b). McrBC was used because it recognizes specific nucleotide sequences containing two methylated cytosines. A comparison of band intensities shown in Figure 2b (lanes 13–16 indicated by a white arrowhead) indicated that the proviral PVCV loci of genomic DNA in leaves of 1‐ and 2‐month‐old plants were methylated whereas those in 3‐month‐old plants were not. Furthermore, the result also indicated that both putative circular and linear forms of episomal PVCV DNAs, which increased substantially in the 4‐month‐old plant, were not methylated (compare the band pattern and intensity in lane 12 with those in lane 16 in Figure 2b; both lanes indicate undigested episomal DNAs).

Furthermore, the methylation state of the PVCV promoter region was examined using the bisulfite sequencing method. The CG and CHG sites in the PVCV promoter region in leaves of the 1‐month‐old plant were highly methylated, but almost all cytosines in the PVCV promoter region in the 3‐month‐old plant were not methylated (Figure S3). However, nucleotide sequences of these PCR clones revealed that most analyzed clones obtained from the 3‐month‐old plant were derived from episomal PVCV DNAs (Figure S3). These results indicate that the promoter region of episomal PVCV DNAs was not methylated (Figure S3), which is consistent with the results using McrBC digestion (Figure 2b) and a previous report (Richert‐Pöggeler *et al.*, 2003).

**Figure 3:**
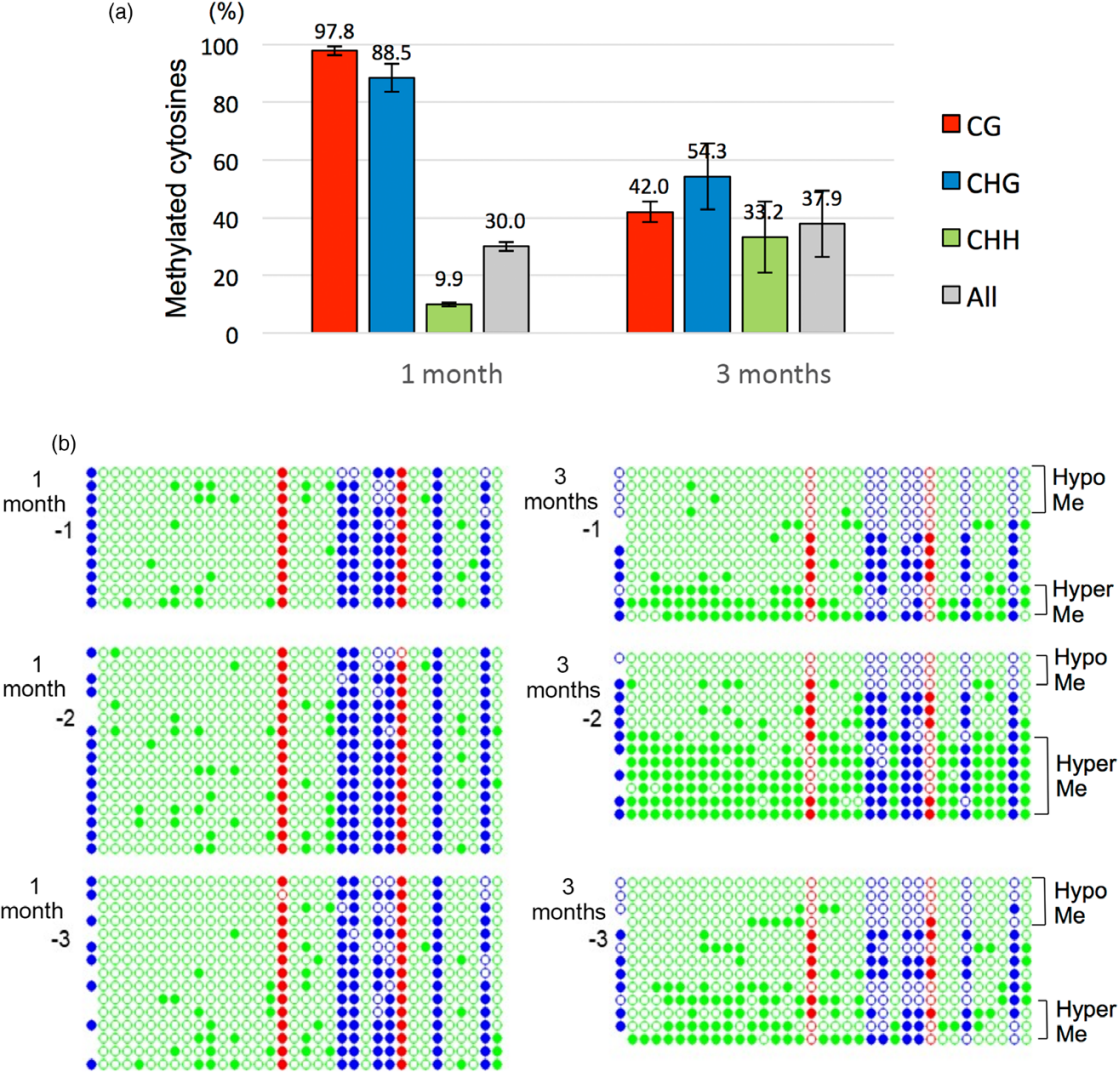
Methylation states of the promoter region of proviral petunia vein clearing caulimovirus (PVCV) in leaves analyzed using the bisulfite sequencing method. Genomic DNAs were prepared from three independent 1‐month‐old plants (1m‐1, 1m‐2, and 1m‐3) and three independent 3‐month‐old plants (3m‐1, 3m‐2, and 3m‐3), and treated using a bisulfite reagent. The promoter region of proviral PVCV was amplified by PCR with provirus‐specific primers (Table S3) and sequenced. (a) The ratios of methylated cytosines in CG (red), CHG (blue), and CHH (green) sites were calculated from sequencing data of *c.* 30 PCR clones. (b) Detailed presentation of methylated cytosines that are indicated as coloured circles. Filled colour circles, red (CG), blue (CHG), and green (CHH), indicate methylated cytosines, and open circles indicate unmethylated cytosines.

To avoid interference of episomal DNA and to accurately analyze the methylation levels of proviral promoter regions in 1‐ and 3‐month‐old plants, bisulfide sequencing analysis was performed using PCR primers that are specific to the proviral PVCV sequence (Figure S1). Sequencing results showed that CG and CHG sites of all PCR clones derived from leaves of 1‐month‐old plants were highly methylated (nearly 100%), but their CHH sites were only slightly methylated (*c.* 10%) (Figure 3a). In contrast, three types of PCR clones derived from proviral PVCV sequences with different methylation states, namely: (a) clones containing barely methylated CG and CHG sites (hypomethylation), (b) clones containing highly methylated CG and CHG sites but slightly methylated CHH sites, and (c) clones containing highly methylated CG, CHG, and CHH sites (hypermethylation), were found in leaves of 3‐month‐old plants (Figure 3b). These results indicated that the methylation levels at CG and CHG sites were not maintained in some of 50 PVCV loci in aged plants, generating some hypomethylated PVCV loci in leaves of 3‐month‐old plants (Figure 3b). These results also suggest that PVCV transcripts were actively transcribed from PVCV loci with unmethylated CG and CHG sites (Figure 2a), and then episomal PVCV DNAs were generated from them (Figure 2b). Once the episomal PVCVs appeared, unmethylated episomal DNA can produce a huge amount of PVCV transcripts (Table S1 and Figure S2).

To examine whether DNA methylation was altered at other loci in the petunia genome during plant ageing, methylation states of promoter regions of *CHS‐A1* and *CHS‐A2* genes, which are duplicated *CHS‐A* genes present in the star‐type petunia cultivar (Morita *et al.*, 2012), were analyzed. In the promoter region of the *CHS‐A2* gene, *c.* 10–20% of cytosines at CG and CHG sites in both DNA samples prepared from 1‐ and 3‐month‐old plants were methylated (Figure S4). In the promoter region of the *CHS‐A1* gene, *c.* 60% of cytosines at CG and CHG sites in both DNA samples were methylated (Figure S5). The highly methylated region contains a short transposon‐like insertion (Morita *et al.*, 2012; Kon and Yoshikawa, 2014). Therefore, the locally high level of DNA methylation may be associated with the methylation mechanism against transposons. An age‐dependent decrease in the frequency of DNA methylation at CG and CHG sites was not observed in both promoter regions. Rather, methylation at CHH sites in a short transposon‐like insertion in the promoter region of the *CHS‐1A* gene increased as plants aged (Figure S5).

### **Induction of** ***de novo*** **methylation at CHH sites in the proviral PVCV sequences as the host plant aged**

Some proviral PVCV promoters from 3‐month‐old plants showed highly methylated CHH sites as well as CG and CHG sites (Figure 3b). The CHH sites in the hypermethylated promoter region were inferred as being *de novo* methylated because the CHH methylations in the same region of 1‐month‐old plants were slightly methylated (*c.* 10%; Figure 3b). Furthermore, both 21‐nt and 24‐nt siRNAs derived from the promoter and coding regions of PVCV were detected in 3‐ and 4‐month‐old plants, and they accumulated more in leaves of 4‐month‐old plants than in those of 3‐month‐old plants (Figure 2c). These results suggest that siRNAs were produced from PVCV transcripts by the host RNA silencing machinery when PVCV was activated in an age‐dependent manner, and then *de novo* RdDM was induced in the promoter regions of proviral PVCV loci to suppress the activation of PVCV (Figure 3b). Namely, the suppressive process against activated proviruses by RdDM was induced in aged petunia plants. In 4‐month‐old plants, both CG and CHG sites in the promoter region of proviral PVCV were highly methylated (proximately 90%), and CHH sites were also considerably methylated (*c.* 25%, Figure S6), supporting the notion that *de novo* RdDM was induced against activated PVCV. However, as shown in Figures 2b (McrBC digestion) and S3 (bisulfite sequencing), episomal DNAs were not methylated even in aged plants, suggesting that host RNA silencing did not function on the episomal PVCV DNAs even in the 3‐ and 4‐month‐old plants.

### Genes involved in ageing, as well as abiotic stress responses, were upregulated in 4‐month‐old plants

Transcriptome (RNA‐seq) analysis was carried out using total RNAs prepared from the leaves of 2‐ and 4‐month‐old plants. Some genes involved in ethylene biosynthesis (1‐aminocyclopropane‐1‐carboxylate oxidase 1 and 4) and signalling (ethylene‐responsive transcription factor 1B) and senescence (senescence‐associated gene 101) were upregulated in 4‐month‐old plants (Table 1). A plant hormone, ethylene, is involved in senescence (ageing). In addition, several genes involved in responses to abiotic stresses, such as oxidation, dehydration, heat, and wounding, were upregulated in 4‐month‐old plants (Table 1). These results indicate that 4‐month‐old plants indeed aged and were exposed to various abiotic stresses during long‐term cultivation. Furthermore, genes involved in responses against pathogen infections, such as a pathogenesis‐related thaumatin superfamily protein and disease resistance protein (TIR‐NBS‐LRR class), were upregulated, suggesting that aged plants responded against the activated PVCV by recognizing it as a pathogen. In contrast, genes encoding histones and a DNA methyltransferase were downregulated in aged plants, which was consistent with the decline of DNA methylation levels in the proviral PVCV loci in the same aged plants.

**Table 1:** Selected upregulated and downregulated genes during long‐term cultivation of petunia

Description	Fold change	*P*‐value	Category
Upregulated			
1‐Aminocyclopropane‐1‐carboxylate oxidase 1	32.045	2.18E‐04	Senescence
Ethylene‐responsive transcription factor 1B	7.271	3.40E‐03	Senescence
DCD (Development and Cell Death) domain protein	4.119	8.45E‐03	Senescence
Senescence‐associated gene 101	2.623	2.60E‐02	Senescence
Pathogenesis‐related thaumatin superfamily protein	25.009	5.63E‐04	Disease resistance
G‐type lectin S‐receptor‐like serine/threonine‐protein kinase	9.346	8.83E‐03	Disease resistance
Disease resistance protein (TIR‐NBS‐LRR class) family	8.406	7.48E‐03	Disease resistance
Copper chaperone for superoxide dismutase	3.159	7.66E‐03	Stress (oxidation)
Glutathione *S*‐transferase TAU 19	15.714	3.33E‐02	Stress (oxidation)
Wound‐induced protein	6.121	1.00E‐02	Stress (wounding)
Temperature‐induced lipocalin	5.685	1.71E‐02	Stress (heat)
Heat shock transcription factor A2	4.647	2.23E‐02	Stress (heat)
Protein DEHYDRATION‐INDUCED 19 homolog 4	3.546	3.89E‐04	Stress (dehydration)
Dicer‐like 2	3.842	1.22E‐01	RNA silencing
Argonaut family protein (AGO2)	7.339	2.30E‐03	RNA silencing
Downregulated			
Histone H2B.10	−8.822	1.45E‐03	Histone
Histone H2A 12	−3.508	4.31E‐02	Histone
Lysine‐specific histone demethylase 1 homolog 1	−3.564	3.97E‐03	Histone
DNA (cytosine‐5)‐methyltransferase 1	−2.198	2.51E‐02	DNA methylation
DNA repair and recombination protein RadA	−4.281	2.93E‐02	Stability of DNA
Argonaut family protein (AGO10)	−3.664	6.05E‐03	RNA silencing
Double‐stranded RNA‐binding protein 4	−2.517	8.02E‐02	RNA silencing
DNA‐directed RNA polymerase IV subunit 1	−2.399	1.70E‐02	RNA silencing

RNA‐seq analysis was carried out using total RNAs prepared from the leaves of 2‐ and 4‐month‐old plants. Upregulated and downregulated genes in 4‐month‐old plants are listed.

### Aged plants had blotched flowers

**Figure 4:**
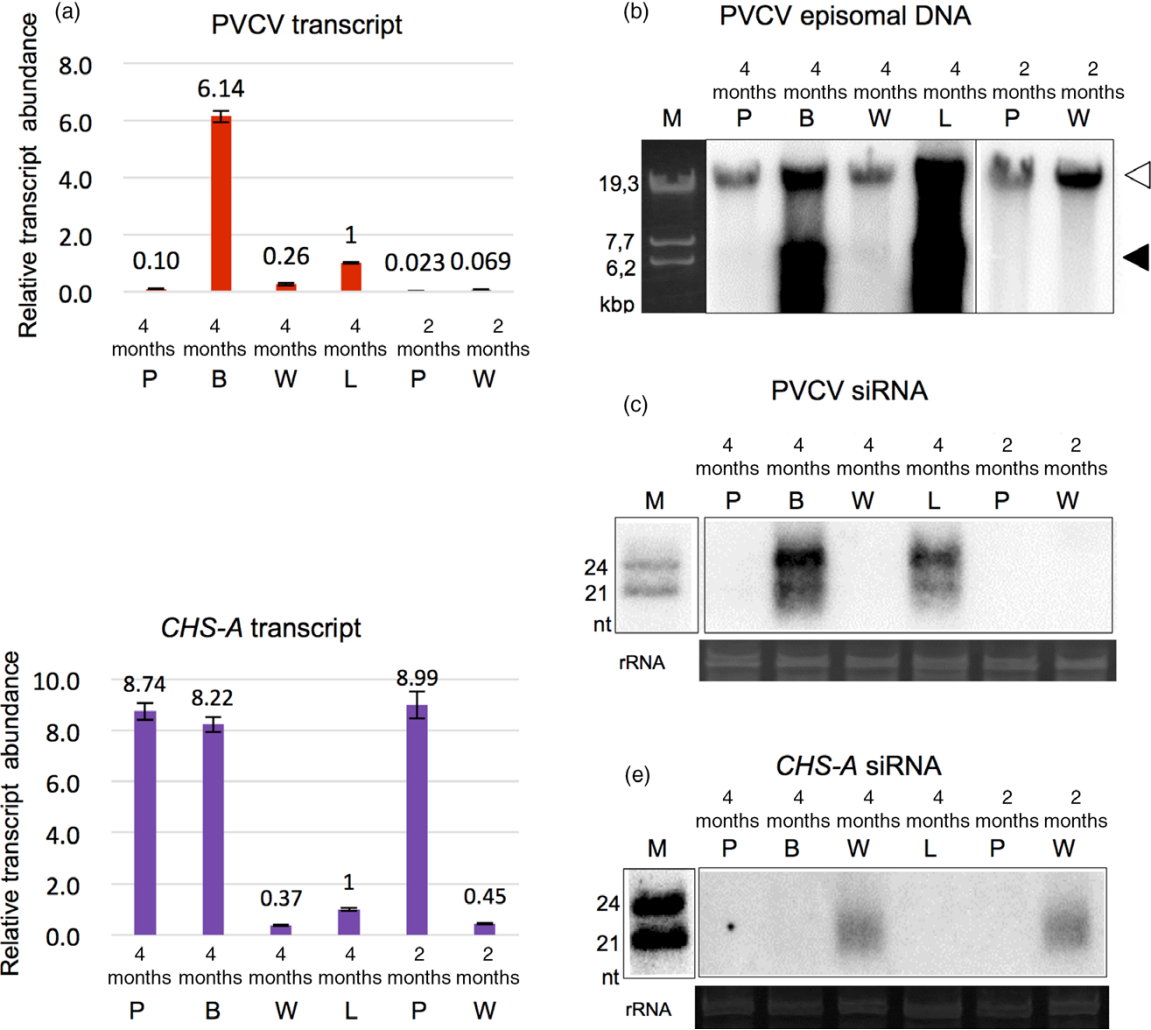
Activation of petunia vein clearing caulimovirus (PVCV) in the blotched region of petals. DNA and total RNAs were isolated from pigmented (4m P), white (4m W) and blotched (4m B) regions of petals and leaves with the vein‐clearing symptom (4m L) of 4‐month‐old plants, and pigmented (2m P) and white (2m W) regions of petals of 2‐month‐old plants. Relative abundance of PVCV transcripts (a) and *CHS‐A* transcripts (d) was determined by qPCR and normalized to the abundance of *Actin7* transcripts. Error bars indicate the standard errors of three biological replications. (b) Proviral (white arrowhead) and episomal (black arrowhead) PVCV DNAs were detected by Southern blot hybridization. PVCV (c) and *CHS‐A* (e) siRNAs were detected by northern blot hybridization. Ribosomal RNAs stained by ethidium bromide are shown as loading controls in (c) and (e). Molecular weight markers of linear dsDNAs (b) and ssRNAs (c and e) are indicated as lane M.

Blotched flowers appeared in 3‐ and 4‐month old plants, in which PVCV was activated (Figure 1). Pigmented blotches were found in the mid‐vein of the white region of petals, where anthocyanin biosynthesis is inhibited by post‐transcriptional *CHS‐A* gene silencing (Koseki *et al.*, 2005). We hypothesized that PVCV activation suppressed *CHS‐A* gene silencing, and then anthocyanin biosynthesis recovered in the white mid‐vein (PTGS) area of petals. The accumulation of PVCV transcripts and episomal DNAs was examined in pigmented (rose), white, and blotched regions of petals by qPCR and Southern blot analyses: both nucleic acids were detected only in the blotched region of petals (lane 4m B in Figure 4a,b) as well as leaves with a vein‐clearing symptom (lane 4m L in Figure 4a,b). In leaves with a vein‐clearing symptom in 4‐month‐old plants, episomal DNAs were detected in the mid‐vein region (lane 4m V in Figure S7) and to a lesser extent, in other regions (lane 4m O in Figure S7) as well.

The accumulation of *CHS‐A* transcripts and siRNAs was examined by qPCR and northern blot analyses to determine whether silencing of the *CHS‐A* gene occurred. As reported previously (Koseki *et al.*, 2005; Kasai *et al.*, 2013), *CHS‐A* transcript barely accumulated in the white region (Figure 4d) where *CHS‐A* siRNAs were detected (Figure 4e), indicating that *CHS‐A* gene silencing occurred in the white regions of petals. In contrast, the transcript level of *CHS‐A* in the blotched region recovered to its level in the pigmented region (Figure 4d), where no *CHS‐A* siRNAs were detected (Figure 4e). These results suggest that PVCV activation suppressed PTGS to recover *CHS‐A* gene expression and anthocyanin biosynthesis in the mid‐vein of white areas in bicoloured petals.

**Figure 5:**
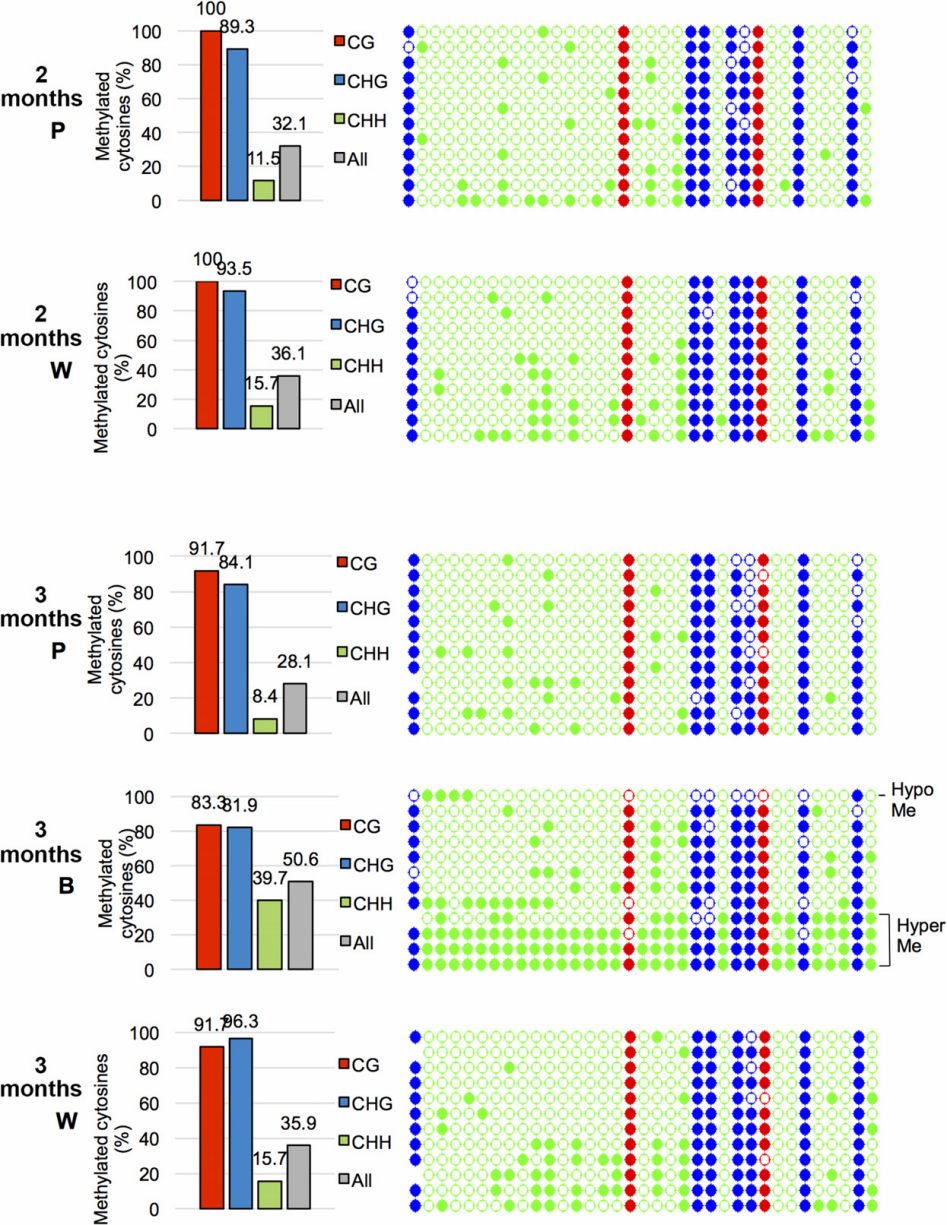
Methylation states of the promoter region of proviral petunia vein clearing caulimovirus (PVCV) in petals. Genomic DNAs were prepared from pigmented (2m P) and white (2m W) regions of petals of 2‐month‐old plants and pigmented (3m P), white (3m W), and blotched (3m B) regions of petals of 3‐month‐old plants, and treated using a bisulfite reagent. The promoter region of proviral PVCV was amplified by PCR with provirus‐specific primers (Table S3) and sequenced. The ratios of methylated cytosines in CG (red), CHG (blue), and CHH (green) sites were calculated from sequencing data of more than 10 PCR clones. Detailed presentation of methylated cytosines that are indicated as coloured circles. Filled colour circles, red (CG), blue (CHG), and green (CHH), indicate methylated cytosines, and open circles indicate unmethylated cytosines.

To determine whether alteration of DNA methylation level in the proviral PVCV loci occurred in petals, the methylation state of the PVCV promoter region was examined in the pigmented, white, and blotched regions of petals of 2‐ and 3‐month‐old plants. Bisulfite sequencing results showed that CG and CHG sites of all PCR clones derived from the pigmented and white regions of petals of both 2‐ and 3‐month‐old plants were highly methylated (90–100%). However, their CHH sites were only slightly methylated (*c.* 10%) (Figure 5). These results were similar to those observed in the leaves of 1‐month‐old plants (Figure 3). In contrast, the blotched regions of petals of 3‐month‐old plants showed less CG and CHG methylation (*c.* 80%), and greater CHH methylation (*c.* 40%, Figure 5) than the pigmented and white regions. These results indicated that the methylation state of the PVCV promoter region in the blotched region of petals was similar to that in the leaves of 3‐ and 4‐month‐old plants (Figures 3 and S6). Therefore, in the blotched petals as well as symptomatic leaves in aged plants, the decrease in DNA methylation level at both CG and CHG sites in the promoter region of proviral PVCV loci was associated with the activation of PVCV. Furthermore, this activation induced *de novo* RdDM. This conclusion is strongly supported by the result in which both 21‐ and 24‐nt siRNAs derived from the promoter region of PVCV were detected in the blotched region of petals (Figure 4c) as well as on the leaves of 4‐month‐old plants (Figure 2c). Furthermore, these results suggested that the PVCV episomal DNAs detected in petals did not move from the leaf veins but emerged from proviruses in the mid‐vein cells of petals.

In cultivars Carnival Red‐Star, Carpet Blue‐Star, and Baccarat Rose‐Picotee, blotched flowers also appeared when plants aged (Figure S8). ‘Picotee’ displays bicoloured flowers with red centres and white margins, which reflect a spatial pattern of anthocyanin biosynthesis distinct from that of star‐type cultivars (Figure S8). Different bicoloured flowers in different cultivars are generated through PTGS of the same duplicated *CHS‐A* genes (Morita *et al.*, 2012). Therefore, the blotched flowers of ‘Picotee’ were also likely to have been induced by the activation of PVCV.

### Detection of suppressor activity of RNA silencing in PVCV

**Figure 6:**
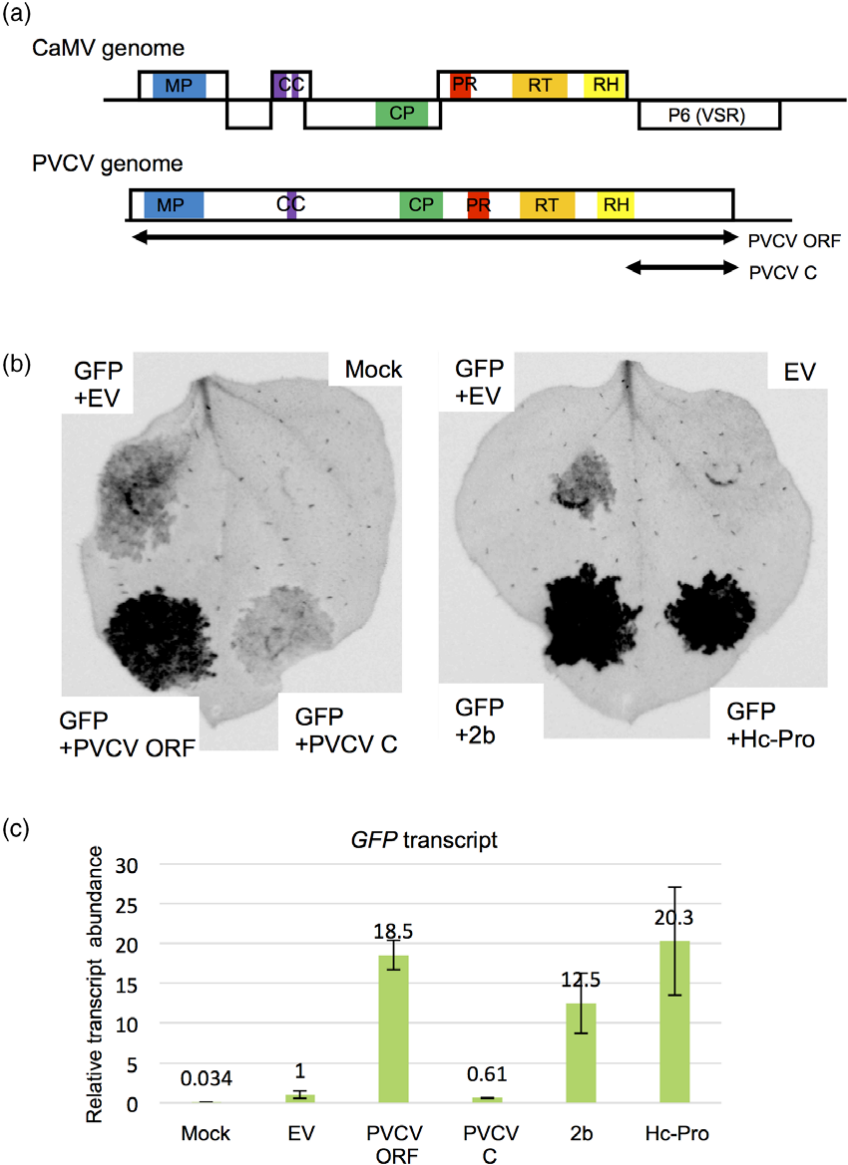
Detection of suppressor activity for RNA silencing from petunia vein clearing caulimovirus (PVCV). (a) Comparison of genomic structures between cauliflower mosaic virus (CaMV) and PVCV. CaMV is a type species in the genus *Caulimovirus* in the family *Caulimoviridae*, and PVCV is a member of the genus *Petuvirus* in this family (Geering and Hull, 2012)*.* The P6 protein encoded by CaMV functions as a viral suppressors of RNA silencing (VSR). Blue, viral movement protein (MP) domain; purple, coiled‐coil (CC) motif; green, coat protein (CP); red, retropepsin (pepsin‐like aspartic protease) (PR) domain; orange, reverse transcriptase (RT) domain; yellow, RNase H1 (RH) domain. Arrows indicate regions analyzed by the agroinfiltration method. (b) Photographs of leaves of *N. benthamiana* 16c plant over‐expressing GFP at 7 days post‐infiltration. Leaves were co‐infiltrated with *Agrobacterium* expressing GFP and PVCV open reading frame (ORF), GFP, and the C‐terminal region of PVCV ORF (PVCV C), GFP, and CMV 2b (positive control), GFP and potyvirus Hc‐Pro (positive control), and GFP and empty vector (EV, negative control). Green fluorescence is shown as black. (c) Relative abundance of *GFP* transcripts in agroinfiltrated regions of *N. benthamiana* leaves was determined by qPCR and normalized to the abundance of *Actin2* transcripts. Error bars indicate the standard errors of three biological replications.

PVCV belongs to the *Caulimoviridae* family, and its dsDNA genome encodes a single long ORF (Geering and Hull, 2012), which encodes a polyprotein homologous to proteins encoded by CaMV, a type species of the *Caulimoviridae* family (Figure 6a)*.* Most plant viruses such as potyviruses and CMV encode VSRs that suppress host RNA silencing, and CaMV also encodes the P6 protein that functions as a VSR (Figure 6a). Thus we expected that PVCV encoded a VSR. The single long ORF sequence encoded by PVCV and its 1115‐nt long 3′‐fragment were cloned into a plasmid vector, and VSR activity was examined by agroinfiltration assays in the leaves of *Nicotiana benthamiana* harbouring over‐expressed green fluorescent protein (GFP)*.* The fluorescence intensities and transcript levels of GFP in agroinfiltrated regions of *N. benthamiana* leaves indicated that the entire ORF of PVCV had VSR activity (Figure 6b,c). Values were comparable with those of CMV’s 2b and potyviral Hc‐Pro proteins that are typical strong VSRs (Figure 6). Conversely, the C‐terminal fragment alone did not exhibit VSR activity (Figure 6), indicating that the protein encoded by this fragment is not sufficient for VSR activity.

## DISCUSSION

**Figure 7:**
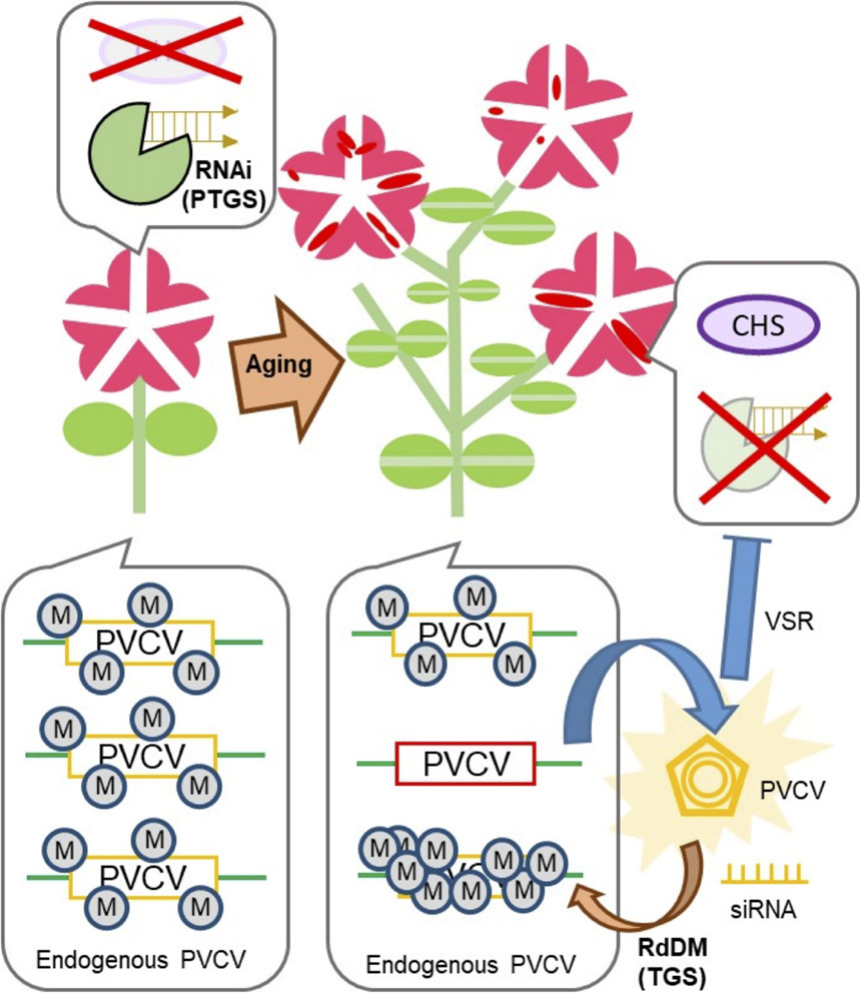
Graphical abstract. PVCV was activated by a decrease in DNA methylation during the long‐term cultivation of host plants, and *CHS* gene silencing was suppressed by a viral suppressors of RNA silencing (VSR) of activated PVCV. In host petunia plants, anthocyanin biosynthesis was restored in the white mid‐vein region of petals, and consequently, blotched flowers appeared. In parallel, *de novo* RdDM was induced, thereby suppressing activated PVCV. M indicates DNA methylation.

In this study, we showed that a decrease in DNA methylation level at both CG and CHG sites in the promoter region of proviral PVCV loci during the long‐term cultivation of host petunia plants is associated with the activation of endogenous pararetrovirus PVCV that encodes a VSR. Consequently, this activation disturbed the floral colour pattern in star‐type petunia cultivars*,* which have bicoloured flowers caused by naturally occurring PTGS (Figure 7). This suggests that maintenance of a high level of CG and/or CHG methylation in the promoter region of proviral PVCV is critical to prevent PVCV activation. The star‐type petunia, together with PVCV, constitute a unique pathosystem that allows visualization of the activation process of an endogenous pararetrovirus (pathogen) by petal colours. Moreover, this pathosystem also allows visualization of the tug‐of‐war between a plant host (RNA silencing) and a pathogen (endogenous pararetrovirus).

In petals, as shown in Figure 4, PVCV was activated only in the blotched region where its transcripts, episomal DNAs, and siRNAs were detected. These results are consistent with the results shown in Figure 5, and indicated that the alteration of methylation state in the PVCV promoter region was detected only in the blotched region. In leaves, because it was difficult to distinguish (isolate) the region (cells) with activated PVCV from other region (cells) with inactivated PVCV, we could not determine whether PVCV was activated only in the mid‐vein regions of leaves or in entire leaves. In petals, in contrast, it was easy to distinguish (isolate) the region (cells) where PVCV was activated, because the blotch was a visible mark where PVCV was activated. We do not know why the PVCV‐activated region (tissue) was more expanded in leaves than in petals. The mechanism for tissue‐ or developmental‐specific activation of PVCV remains unknown.

In animals such as *Caenorhabditis elegans*, *Drosophila melanogaster*, and mammals, there is a connection between activation of transposons and ageing (Wood and Helfand, 2013; Orr, 2016; Wood *et al.*, 2016). In young animals, transposons are completely repressed both transcriptionally and post‐transcriptionally by RNA silencing pathways. As animals age, these genetic surveillance mechanisms break down and become less effective, leading to the loss of heterochromatin structure, and transposon mobilization (activation) (Wood and Helfand, 2013; Orr, 2016). Our observation that an endogenous pararetrovirus, PVCV, is activated as plants age may be a phenomenon similar to the age‐dependent activation of transposons in animals (Wood and Helfand, 2013; Orr, 2016).

DNA methylation and demethylation are involved in epigenetic regulation of gene expression. DNA demethylation is induced by environmental stresses in maize and tobacco and by ageing in *Acacia mangium* (Steward *et al.*, 2002; Baurens *et al.*, 2004; Wada *et al.*, 2004). In this study, as petunia plants were grown in a controlled growth room, they were thought to be grown with few stresses in comparison with field‐grown plants, suggesting that the alteration of DNA methylation level, PVCV activation, and occurrence of blotched flowers, were induced primarily by ageing. However, the results of transcriptome analysis showed that genes involved in senescence and ageing, as well as responses to abiotic stresses, such as heat, drought, and wounding, were upregulated in 4‐month‐old plants, suggesting that aged plants might be exposed to various abiotic stresses during long‐term cultivation. Therefore, PVCV might be activated by the accumulation of various abiotic stresses during long‐term cultivation.

Both hyper‐ and hypomethylated promoter sequences of proviral PVCVs (Figures 3, 5 and S6), as well as 24‐nt siRNAs derived from them (Figures 2c and 4c), were detected in leaves and petals of 3‐ and 4‐month‐old plants. These results indicated that the host plants activated RNA silencing, including 24‐nt siRNA production and *de novo* RdDM to counter the age‐induced PVCV activation. The 24‐nt siRNA production and *de novo* RdDM are crucial processes for suppressing activated transposons in *A. thaliana* (Mirouze *et al.*, 2009; Marí‐Ordóñez *et al.*, 2013). It is reasonable to assume that an endogenous virus in petunia, which is similar to a retrotransposon, is also re‐silenced by the host RdDM machinery. Concurrently, transcriptome analysis indicated that some genes involved in defence responses against pathogen infections were upregulated in aged plants (Table 1), suggesting that aged plants recognized activated PVCV as a pathogen and likely responded to suppress it by the R‐gene‐mediated resistant mechanism as well as RNA silencing via RdDM.

The methylation state of the promoter region of PVCV in 1‐month‐old plants may be unique relative to transposons because both CG and CHG sites were highly methylated but CHH sites were only slightly methylated (Figure 3). This result suggests that *de novo* methylation via 24‐nt siRNAs (RdDM) barely occurred in the promoter region of proviral PVCV in 1‐month‐old plants. This inference is consistent with the result in which no small RNAs derived from the promoter region of PVCV were detected in 1‐month‐old plants (Figure 2c). In the transposon‐like insertion of the *CHS‐A1* promoter, in contrast, both CG and CHG sites were highly methylated, and CHH sites were also considerably methylated. This result is consistent with previous reports indicating that RdDM occurs on almost all transposon sequences (Castel and Martienssen, 2013; Matzke and Mosher, 2014). As RdDM at CHH sites is likely to be critical to prevent the activation of parasitic nucleic acids such as transposons and endogenous viruses, the results of the present study (Figures 3 and 5) suggest that proviral PVCV sequences that are unmethylated at CHH sites tend to be activated more frequently than transposons that are methylated at all cytosines.

In Figure 6, we show that PVCV encodes a VSR that has suppressor activity comparable with authentic VSRs such as CMV 2b and potyviral Hc‐Pro. This is the first report showing that an endogenous pararetrovirus encodes a VSR. Surely, novel VSRs will be found from other endogenous pararetroviruses in the near future. Although we expected that a putative VSR was encoded in the 3′‐region of the PVCV ORF based on genome organization of a homologous CaMV (Figure 6a), VSR activity was not detected from the 3′‐fragment of the ORF, suggesting that either a VSR is not encoded by the 3′‐region of this ORF, or the exact processing of a single polyprotein by a proteinase encoded by another region is essential for producing a functional VSR.

Endogenous pararetrovirus sequences are the most commonly found virus sequences and constitute some fractions of repetitive sequences in the genomes of a wide range of angiosperms (Chabannes and Iskra‐Caruana, 2013; Becher *et al.*, 2014). Most of these sequences no longer propagate in the host, but novel endogenous pararetroviruses may be activated to propagate as host plants age, similar to what PVCV does. However, whereas the activation of PVCV is easily detectable due to the appearance of blotches in bicoloured flowers of petunia, the detection of activated pararetroviruses may be difficult if symptoms of host plants are not so prominent.

It is well known in mammals, including humans, that endogenous retrovirus and non‐retroviral virus sequences are beneficial to the host, such as providing virus resistance (Jern and Coffin, 2008; Horie *et al.*, 2010; Stoye, 2012). Similarly, endogenous pararetrovirus sequences can also contribute virus resistance to the host plant through TGS (RdDM) and PTGS (RNAi) mechanisms (Mette *et al.*, 2002; Noreen *et al.*, 2007; Bertsch *et al.*, 2009; Chabannes and Iskra‐Caruana, 2013). Consequently, these endogenous pararetrovirus sequences are likely to be retained by positive‐selection pressure. Furthermore, in the combination between star‐type petunia cultivars and PVCV, the maintenance of PVCV on the host and its activation during the host’s ageing are probably of specific biological significance. The blotched flowers produced by PVCV activation are likely to be more resistant to herbivores than flowers without blotched regions due to the antiherbivore function of the pigment anthocyanin (Johnson *et al.*, 2008).

## Experimental procedures

### Plant materials and growth conditions

Petunia (*P. hybrida*) and *N. benthamiana* plants were grown in pots in a controlled‐environment room under the following conditions: 40–50 μmol m^−2^ sec^−1^, 16 h light and 8 h dark, 24°C. Seeds of *N. benthamiana* line 16c were kindly provided by David Baulcombe, the Sainsbury Laboratory, Norwich, UK. Commercially available seeds of petunia, Rondo Rose‐Star, Carnival Red‐Star, Carpet Blue‐Star, and Baccarat Rose‐Picotee were purchased from seed companies (TAKII and SAKATA SEED, Japan).

### Total RNA extraction

Total RNA was extracted from young leaves or petals of 1‐ to 4‐month‐old petunia plants using TRIzol reagent following the manufacturer’s protocol (Thermo Fisher Scientific, Waltham, MA, USA).

### Quantitative real‐time PCR (qPCR)

cDNAs were produced from total RNAs by a PrimeScript RT reagent kit with gDNA Eraser (TaKaRa, Shiga, Japan), and quantitative real‐time PCR (qPCR) was performed by a Thermal Cycler Dice Real‐Time System with a SYBR Premix Ex Taq II kit (TaKaRa, Japan). Primers for qPCR were designed using the Primer3Plus program (http://www.bioinformatics.nl/cgi‐bin/primer3plus/primer3plus.cgi/) with Sol Genomics Network (https://solgenomics.net/). Primers are listed in Table S3.

### Detection of small RNAs (sRNAs)

Approximately 5 μg of total RNA was electrophoresed in 18% denaturing polyacrylamide gels containing 1× TBE buffer [89 mm Tris (pH 8.3), 89 mm boric acid, 2 mm EDTA] and 7 m urea, and blotted onto a nylon membrane (Zeta‐Probe, Bio‐Rad, Hercules, CA, USA) by electroblotting (1.0 mA/1.0 cm^2^ membrane for 1 h at 4°C). DNA fragments of PVCV and the *CHS‐A* gene as probes were amplified by PCR, and then probes for siRNA detection were made using the BcaBEST Labeling Kit (TaKaRa) with [α‐^32^P]dCTP. PCR primers are listed in Table S3. Hybridization was carried out in Perfect Hyb Plus hybridization buffer (Sigma‐Aldrich, St. Louis, MO, USA) containing ^32^P‐labelled DNA probe at 50°C for 6 h. Membranes were washed four times in 2× SSC (1× SSC, 0.15 m NaCl, 15 mm sodium citrate) with 0.5% SDS at 50°C for 1 h, and then analyzed by a Typhoon FLA 7000 image analyzer (GE Healthcare, Chicago, IL, USA) (Fukuhara *et al*, 2011).

### McrBC digestion and Southern hybridization

Total genomic DNAs were isolated from petunia leaves or petals following the protocol described by Liu *et al.* (1995). Approximately 4 μg of purified DNA was digested with the restriction endonuclease McrBC (0.3 U/20 μl of the reaction mixture) at 37°C for 20 h, separated by agarose gel electrophoresis, then stained with ethidium bromide. For Southern hybridization, DNA fragments were transferred to a nylon membrane (Zeta‐Probe, Bio‐Rad) by the capillary transfer method. Southern hybridization to detect proviral and episomal PVCV sequences was carried out by the same protocol as siRNA detection except for hybridization and a wash temperature of 65°C.

### Bisulfite sequencing for DNA methylation analysis

High‐molecular‐weight genomic DNAs were isolated from total DNAs extracted from petunia leaves or petals by low‐melting point agarose gel electrophoresis. Bisulfite conversion of purified high‐molecular‐weight DNA of 200 ng was performed using the Epitect Bisulfite kit (Qiagen Inc., Hilden, Germany) according to the manufacturer's instructions. The sense strands of bisulfite‐modified DNA of the PVCV promoter region were amplified by PCR using KOD‐Multi & Epi (Toyobo, Japan) according to the manufacturer’s instructions. PCR primers are listed in Table S3. The amplified PCR products were cloned into the pUC118 *Hin*cII/BAP vector using DNA Ligation Kit Ver.2.1 (TaKaRa) according to the manufacturer's instructions. The ligation products were transformed into *Escherichia coli* strain DH5α, and the plasmids containing PCR fragments were purified from a single transformed *E. coli* colony. Sanger sequencing of cloned plasmids was performed using Applied Biosystems 3130x1 Genetic Analyzers with ABI PRISM^®^ BigDye^®^ Terminator v3.1 Cycle Sequencing Kits and Sequencing Analysis Software Version 5.2 (Applied Biosystems, Waltham, MA, USA). Finally, the distributions and ratios of methylated cytosine residues were analyzed using Kismeth software (Gruntman *et al.*, 2008). The same experiment using purified plasmids containing the PVCV promoter region was performed to calculate bisulfite conversion efficiency, and it was 98.75%.

### ***Agrobacterium*** **‐mediated transient expression assay (agroinfiltration)**

DNA fragments of the full‐length and C‐terminal region of the PVCV ORF were amplified from petunia genomic DNA by PCR with PrimeSTAR^®^ Max DNA Polymerase (TaKaRa), and then cloned between the CaMV 35S promoter and the NOS terminator of the binary vector pRI201‐AN (Takara) using the In‐Fusion^®^ HD Cloning kit (TaKaRa). PCR primers are listed in Table S3. The construction of pRI201‐AN containing authentic VSRs, potyviral Hc‐Pro, and CMV 2b, and a binary vector expressing the *GFP* gene (pGA482) were described previously (Takahashi *et al.*, 2012). Binary plasmids were introduced into *Rhizobium radiobacter* (*Agrobacterium tumefaciens*) LBA4404 (TaKaRa) by the electroporation method with MicroPulser™ (Bio‐Rad). RNA silencing‐suppressor activities of proteins encoded by PVCV were investigated in GFP‐expressing *N. benthamiana* line 16c by an *Agrobacterium*‐mediated transient expression assay (agroinfiltration) (Yaegashi *et al.*, 2007). Green fluorescence in leaves of *N. benthamiana* line 16c was measured using the imaging analyzer LAS‐3000 (FUJIFILM, Tokyo, Japan).

### Transcriptome analysis

A library preparation for RNA‐sequencing (RNA‐seq) was performed using the TruSeq stranded mRNA sample prep kit (Illumina, San Diego, CA, USA) according to the manufacturer’s instructions. Whole transcriptome sequencing was applied to the RNA samples with the use of an Illumina HiSeq 2500 platform in a 75‐base single‐end mode. Illumina Casava version 1.8.2 software was used for base calling. Sequenced reads were mapped to the petunia genome using TopHat version 2.0.13 in combination with Bowtie2 version 2.2.3 and SAMtools version 0.1.19. The number of fragments per kilobase of exon per million mapped fragments (FPKMs) was calculated using Cuffnorm version 2.2.1 (Trapnell *et al.*, 2012). As the genome of *P. hybrida* is an amphidiploid of *Petunia axillaris* and *Petunia inflata*, genome data were prepared to merge these genome datasets (*P. axillaris*, v.1.0.1; *P. inflata*, v.1.6.2; Sol Genomic Network https://solgenomics.net/).

Sequenced reads resulting from transcriptome analysis were mapped against the PVCV genome (episomal DNA sequence, U95208.2) using Bowtie2 version 2.3.3.1 (Langmead and Salzberg, 2012). After converting them into .bam files, the status of mapped reads was visualized using an Integrative Genomics Viewer (Robinson *et al.*, 2011). The aligned reads were validated and counted, and to determine whether these were derived from episomal or proviral DNA referring single nucleotide polymorphisms (SNPs), and they were assessed by bisulfite sequencing.

## ACCESSION NUMBER

The datasets generated in the current study are available in GSE145373. NCBI GEO (http://www.ncbi.nlm.nih.gov/geo/).

## AUTHOR CONTRIBUTIONS

KK, HM, HK, HT, and TF designed the research. KK performed northern and Southern hybridization, qPCR, bisulfite sequencing and agroinfiltration. MT performed transcriptome analysis. KK, MT, AK, and TF wrote the paper. All authors read and approved the final manuscript.

## CONFLICT OF INTEREST

The authors declare no conflict of interests.

### OPEN RESEARCH BADGES

This article has earned an Open Data Badge for making publicly available the digitally shareable data necessary to reproduce the reported results.

This article has earned an Open Materials Badge for making publicly available the components of the research methodology needed to reproduce the reported procedure and analysis.

## Supporting information


**Figure S1.** Difference in nucleotide sequences between proviral and episomal PVCVs.
**Figure S2.** Ratios of proviral and episomal PVCV transcripts (reads) in RNA‐seq datasets prepared from three independent 4‐month‐old plants of petunia cv Rondo Rose‐Star.
**Figure S3.** Methylation states of the promoter region of PVCV analyzed using the bisulfite sequencing method.
**Figure S4.** Methylation states of the promoter region of the *CHS‐A2* gene analyzed using the bisulfite sequencing method.
**Figure S5.** Methylation states of the promoter region of the *CHS‐A1* gene analyzed using the bisulfite sequencing method.
**Figure S6.** Methylation states of the promoter region of proviral PVCV analyzed using the bisulfite sequencing method.
**Figure S7.** Detection of episomal PVCV DNA in leaves.
**Figure S8.** Occurrence of blotched flowers as the host plant ages.

**Table S1.** Ratios of PVCV reads per total reads in RNA‐seq datasets.
**Table S2.** Ratios of proviral and episomal PVCV sequences in RNA‐seq datasets prepared from three independent 4‐month‐old plants of petunia cv Rondo Rose‐Star.
**Table S3.** Sequences of oligo DNAs.
