# Supplementary material for: Disturbance of floral colour pattern by activation of an endogenous pararetrovirus, petunia vein clearing virus, in aged petunia plants
Source: Plant J. 2020 Apr 3;103(2):497–511. doi: 10.1111/tpj.14728 (PMC7496347; doi:10.1111/tpj.14728)
Supplement: Supplementary file 1 — Figure S1. Difference in nucleotide sequences between proviral and episomal PVCVs. Figure S2. Ratios of proviral and episomal PVCV transcripts (reads) in RNA‐seq datasets prepared from three independent 4‐month‐old plants of petunia cv Rondo Rose‐Star. Figure S3. Methylation states of the promoter region of PVCV analyzed using the bisulfite sequencing method. Figure S4. Methylation states of the promoter region of the CHS‐A2 gene analyzed using the bisulfite sequencing method. Figure S5. Methylation states of the promoter region of the CHS‐A1 gene analyzed using the bisulfite sequencing method. Figure S6. Methylation states of the promoter region of proviral PVCV analyzed using the bisulfite sequencing method. Figure S7. Detection of episomal PVCV DNA in leaves. Figure S8. Occurrence of blotched flowers as the host plant ages. [file TPJ-103-497-s001.pdf]

Figure S1

```
                                                                              1
rondo_episomal     AGTTTGTCTAGTCTACTTCTGTCTTTATTAAAAGTAAGGAATCTCTTGCTTTTTCTATTA 60
rondo_provirus     AGTTTGTCTAGTCTACTTCTGTCTTTATTAAAAGTAAGGAATCTCTTGCTTTTTCTATTT 60
episomal           AGTTTGTCTAGTCTACTTCTGTCTTTATTAAAAGTAAGGAATCTCTTGCTTTTTCTATTA 60
provirus           AGTTTGTCTAGTCTACTTCTGTCTTTATTAAAAGTAAGGAATCTCTTGCTTTTTCTATTT 60
                   *********************************************************** 

                                                                     NS_F
rondo_episomal     TGGCATTTTGTAATAATGCTCTGTCCACTATCGAGTCTGTATTATTAAGTGTGATAAGTG 120
rondo_provirus     TGGCATTTTGTAATAATGCTCTGTCCACTATCGAGTCTGTATTATTAAGTATGATAAGTG 120
episomal           TGGCATTTTGTAATAATGCTCTGTCCACTATCGAGTCTGTATTATTAAGTGTGATAAGTG 120
provirus           TGGCATTTTGTAATAATGCTCTGTCCACTATCGAGTCTGTATTATTAAGTGTGATAAGTG 120
                   ************************************************** *********

                                                   2
rondo_episomal     TGCTGTCATGATGTAAGTGCTTACGTCTTGAGTCATAGAGATTGCCTATATAAGGTAACC 180
rondo_provirus     TGCTGTCATGATGTAAGTGCTTACGTCTTGAGCCATAGAGATTGCCTATATAAGGTAACC 180
episomal           TGCTGTCATGATGTAAGTGCTTACGTCTTGAGTCATAGAGATTGCCTATATAAGGTAACC 180
provirus           TGCTGCCATGATGTAAGTGCTTACGTCTTGAGCCATAGAGATTGCCTATATAAGGTACCC 180
                   ***** ************************** ************************ **

                         PS_F
                      3         4
rondo_episomal     TCATTAGTTGTTT-CGATCAGAACCAACTTTCCTTTGTAAAATATTATCATCAATAAAAT 239
rondo_provirus     TCACTAGTTGTTTTCAGGCAGAACCAACTTTCCTTTGTAAAATATTATCATCAATAAAAT 240
episomal           TCATTAGTTGTTTTCGATCAGAACCAACTTTCCTTTGTAAAATATTATCATCAATAAAAT 240
provirus           TCACTAGTTGTTTTTAGGCAGAACCAACTTTCCTTTGTAAAATATTATCATCAATAAAAT 240
                   *** *********     ******************************************

                                       5                              6
rondo_episomal     GTTTGTGTGAATAACTCTCTCTTGTTTCCAAACTCTCAAAATGGTAGTTCGATCCTAATA 299
rondo_provirus     GTTTGTGTGAATAACTCTCT--TGTTTCCAAACTCTCAAAATGGTAGTTCGGTCCTAATA 298
episomal           GTTTGTGTGAATAACTCTCTCTTGTTTCCAAACTCTCAAAATGGTAGTTCGATCCTAATA 300
provirus           GTTTGTGTGAATAACTCTCT--TGTTTCCAAACTCTCAAAATGGTAGTTCGGTCCTAATA 298
                   ********************  ***************************** ********

                                                 NS_R
rondo_episomal     TTAAATTGAAGGTGATTACTCTATAAGAGTGATTACTGAAGATAGTAGGTCAGATTAAGG 359
rondo_provirus     TTAAATTGAAGGTGATTACTCTATAAGAGTGATTACTGAAGATAGTAGGTCAGATTAAGG 358
episomal           TTAAATTGAAGGTGATTACTCTATAAGAGTGATTACTGAAGATAGTAGGTCAGATTAAGG 360
provirus           TTAAATTGAAGGTGATTACTCTATAAGAGTGATTACTGAAGATAGTAGGTCAGATTAAGG 358
                   ************************************************************

                                              7           8
rondo_episomal     TTATTGACTTGGTATTATGGCTGTATGGTGCCGTGTTAAAGCCCAGTAGTCTAACAA-AA 418
rondo_provirus     TTATTGACTTGGTATTATGGCTGTATGATGCCGTGTTAA-GCCCAGTAGTCTAACAAAAA 417
episomal           TTATTGACTTGGTATTATGGCTGTATGGTGCCGTGTTAAAGCCCAGTAGTCTAACAA-AA 419
provirus           TTATTGACTTGGTATTATGGCTGTATGATGCCGTGTTAA-GCCCAGTAGTCTAACATCAA 417
                   *************************** *********** ****************   **

                                 9          PS_R
rondo_episomal     CATCTGATCTAGTTATCCTCAGTTTAATCAGAATTCTCTTATAGGTTCAATCT 471
rondo_provirus     CATCTGATCTAGTAAGCCTCAGTTTAATCAGAATTCTCTTATAGGTTCAATCT 470
episomal           CATCTGATCTAGTTATCCTCAGTTTAATCAGAATTCTCTTATAGGTTCAATCT 472
provirus           CATCTGATCTAGTTAGCCTCAGTTTAATCAGAATTCTCTTATAGGTTCAATCT 470
                   ************* * *************************************
```

Figure S1. Difference in nucleotide sequences between proviral and episomal PVCVs. The nucleotide sequence of the promoter region of proviral PVCV is different from that of episomal DNA in *P. hybrida* (Richert-Poggeler et al., 2003). The nucleotide sequences of PCR fragments amplified from total RNAs prepared from 4-month-old petunia plants of cv. Rondo Rose-Star were compared with those of proviral and episomal PVCVs deposited in public databases (AY228106 and U95208.2). Thereby, the most similar sequences (clones) with proviral and episomal PVCVs, respectively, were determined. Multiple alignment of these four nucleotide sequences of the promoter region of PVCV as well as the locations of two pairs of PCR primers designed for bisulfite sequencing are shown. One pair is designed to specifically amplify proviral sequences (provirus-specific primers, PS_F and PS_R), and the other is designed to amplify both proviral and episomal sequences (non-specific primers, NS_F and NS_R) (Table S3). The data shown in Figs. 3, 5 and S6 were obtained using provirus-specific primers and the data shown in Fig. S3 were obtained using non-specific primers.

Figure S2

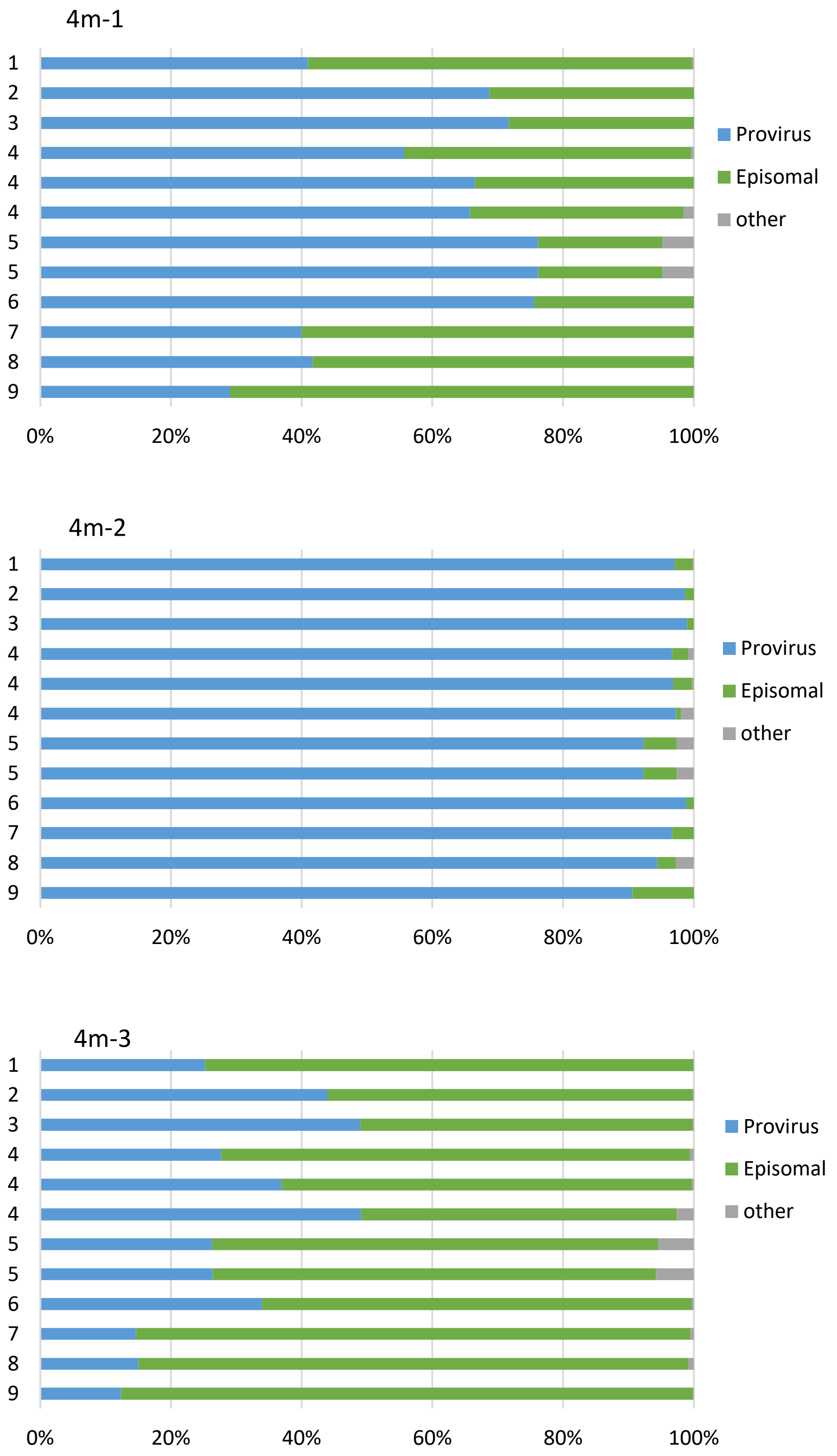

Figure S2. The ratios of proviral and episomal PVCV transcripts (reads) in RNA-seq datasets prepared from three independent 4-month-old plants of petunia cv. Rondo Rose-Star. The ratios were calculated from the number of reads on nine distinguishable sites between proviral and episomal sequences, which are shown in Figure S1, in RNA-seq datasets.

Figure S3

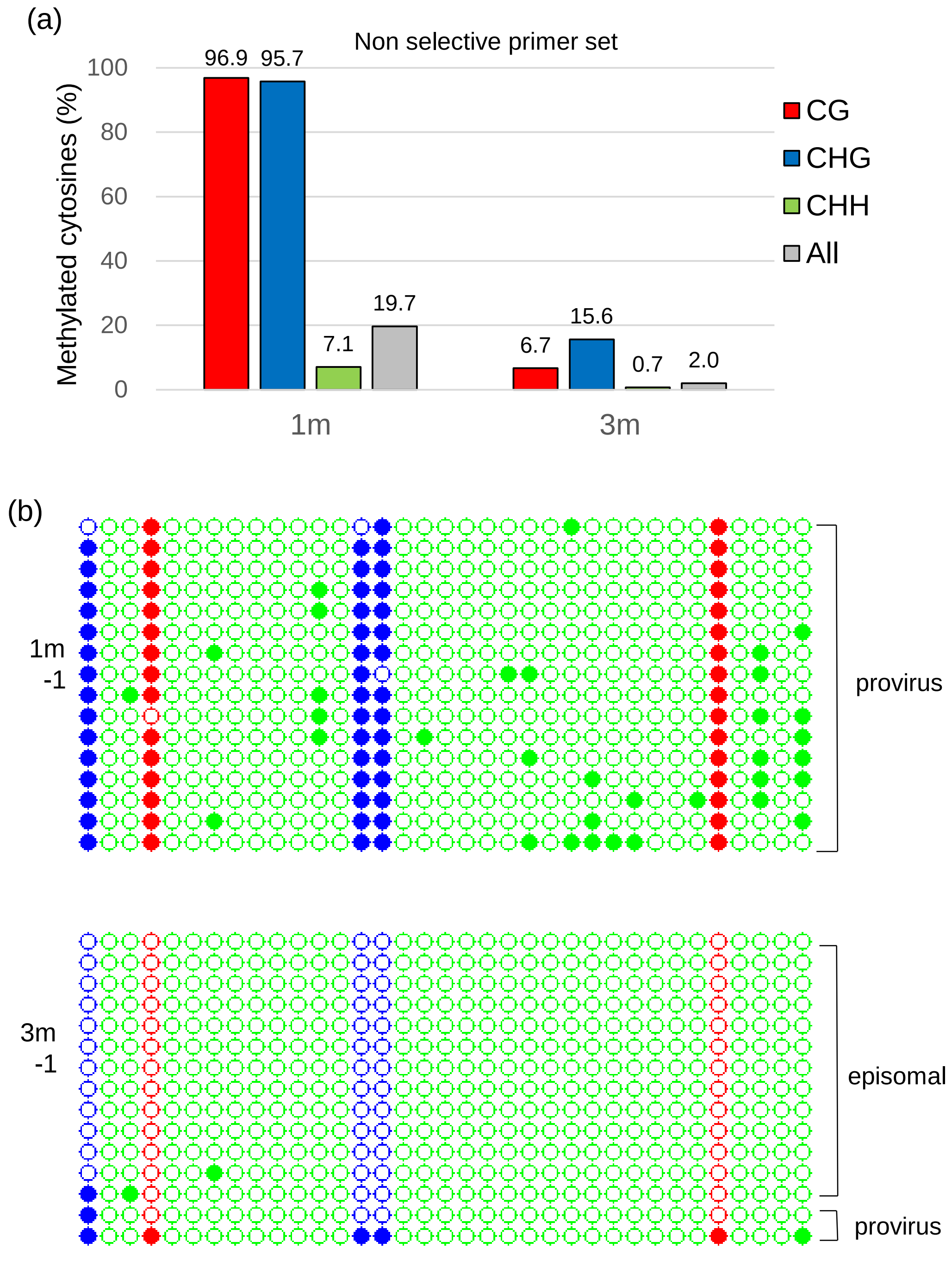

Figure S3. Methylation states of the promoter region of PVCV analyzed using the bisulfite sequencing method. Genomic DNAs were prepared from leaves of three 1-month-old (1m) and three 3-month-old (3m) plants and treated using a bisulfite reagent. The promoter regions of both proviral and episomal PVCV were amplified by PCR with non-specific PVCV primers (NS_F and NS_R in Figure S1 and Table S3), and sequenced. (a) The ratios of methylated cytosines in CG (red), CHG (blue) and CHH (green) sites were calculated from sequencing data of approximately 15 PCR clones. (b) Detailed presentation of methylated cytosines that are indicated as colored circles. Filled color circles, red (CG), blue (CHG), and green (CHH), indicate methylated cytosines, and open circles indicate ummethylated cytosines.

Figure S4

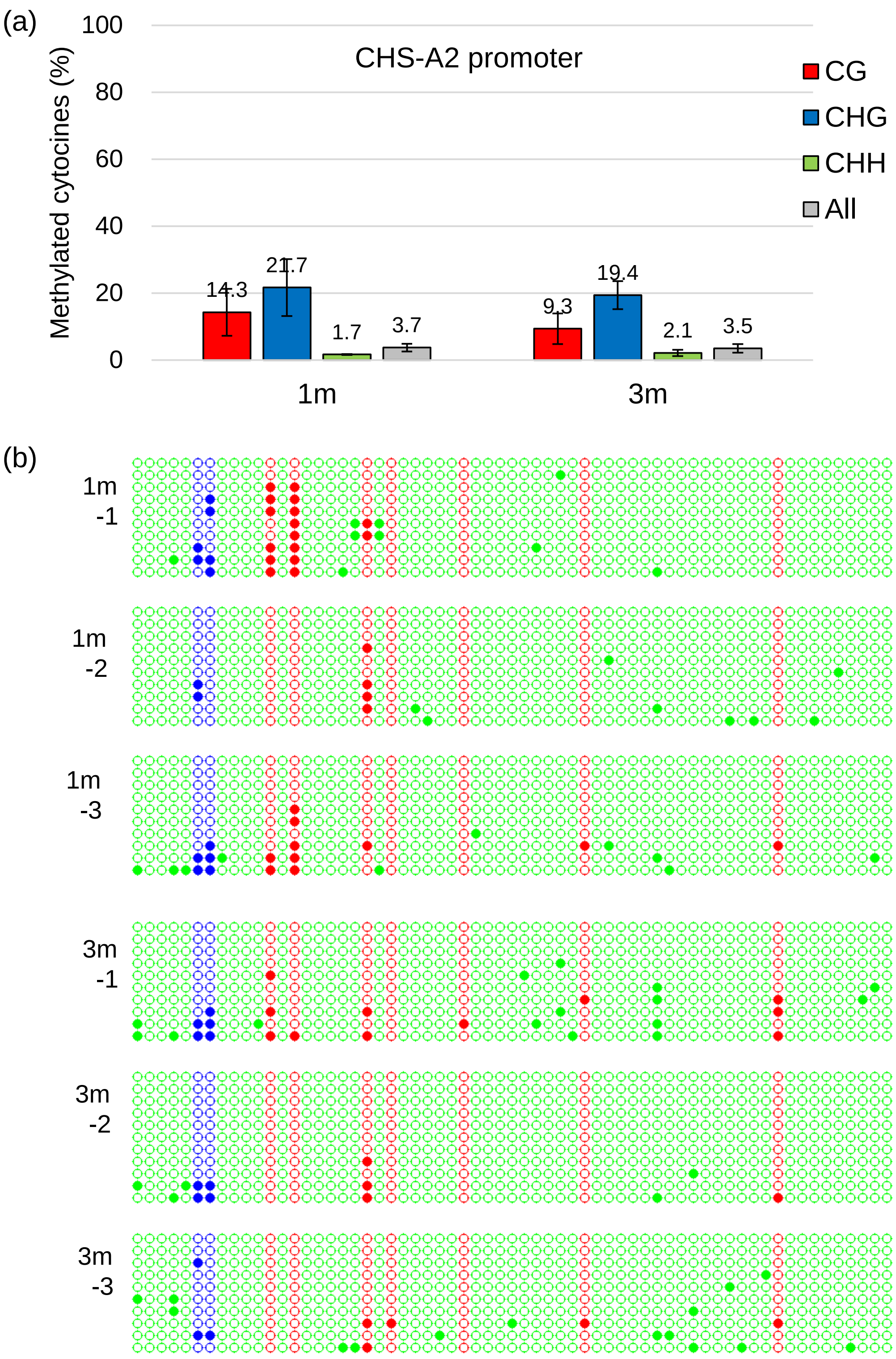

Figure S4. Methylation states of the promoter region of the *CHS-A2* gene analyzed using the bisulfite sequencing method. Genomic DNAs were prepared from leaves of three 1-month-old (1m) and three 3-month-old (3m) plants and treated using a bisulfite reagent. The promoter region of the *CHS-A2* gene was amplified by PCR and sequenced. (a) The ratios of methylated cytosines in CG (red), CHG (blue) and CHH (green) sites were calculated from sequencing data of approximately 30 PCR clones. (b) Detailed presentation of methylated cytosines that are indicated as colored circles. Filled color circles, red (CG), blue (CHG), and green (CHH), indicate methylated cytosines, and open circles indicate unmethylated cytosines.

Figure S5

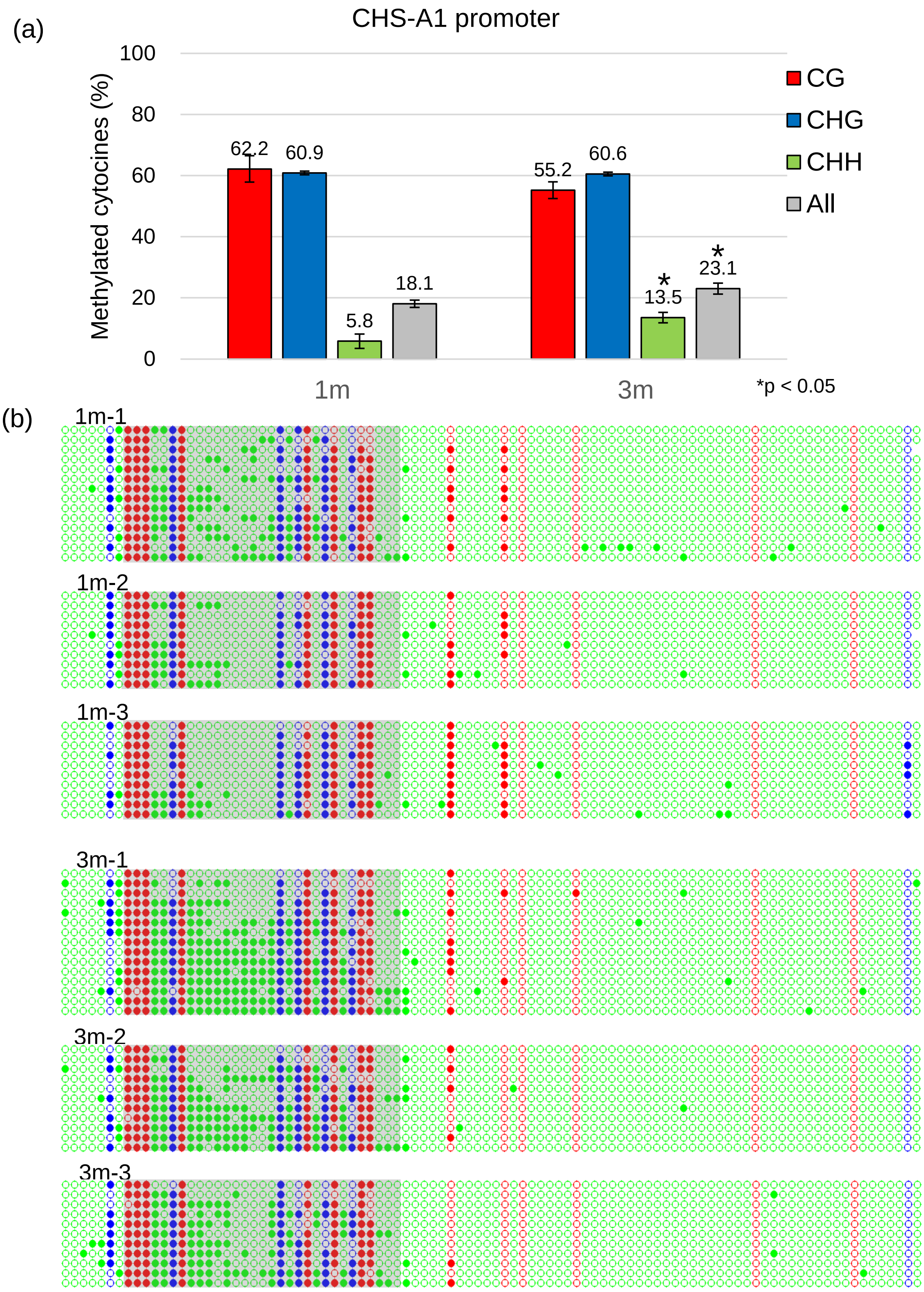

Figure S5. Methylation states of the promoter region of the *CHS-A1* gene analyzed using the bisulfite sequencing method. Genomic DNAs were prepared from leaves of three 1-month-old (1m) and three 3-month-old (3m) plants and treated using a bisulfite reagent. The promoter region of the *CHS-A1* gene was amplified by PCR and sequenced. (a) The ratios of methylated cytosines in CG (red), CHG (blue) and CHH (green) sites were calculated from sequencing data of approximately 30 PCR clones. p-values were determined using the Student's *t*-test. Asterisks (*) indicate significant differences between 1- and 3-month-old plants ($p < 0.05$). (b) Detailed presentation of methylated cytosines that are indicated as colored circles. Filled color circles, red (CG), blue (CHG), and green (CHH), indicate methylated cytosines, and open circles indicate unmethylated cytosines. Gray indicates a short transposon-like insertion.

Figure S6

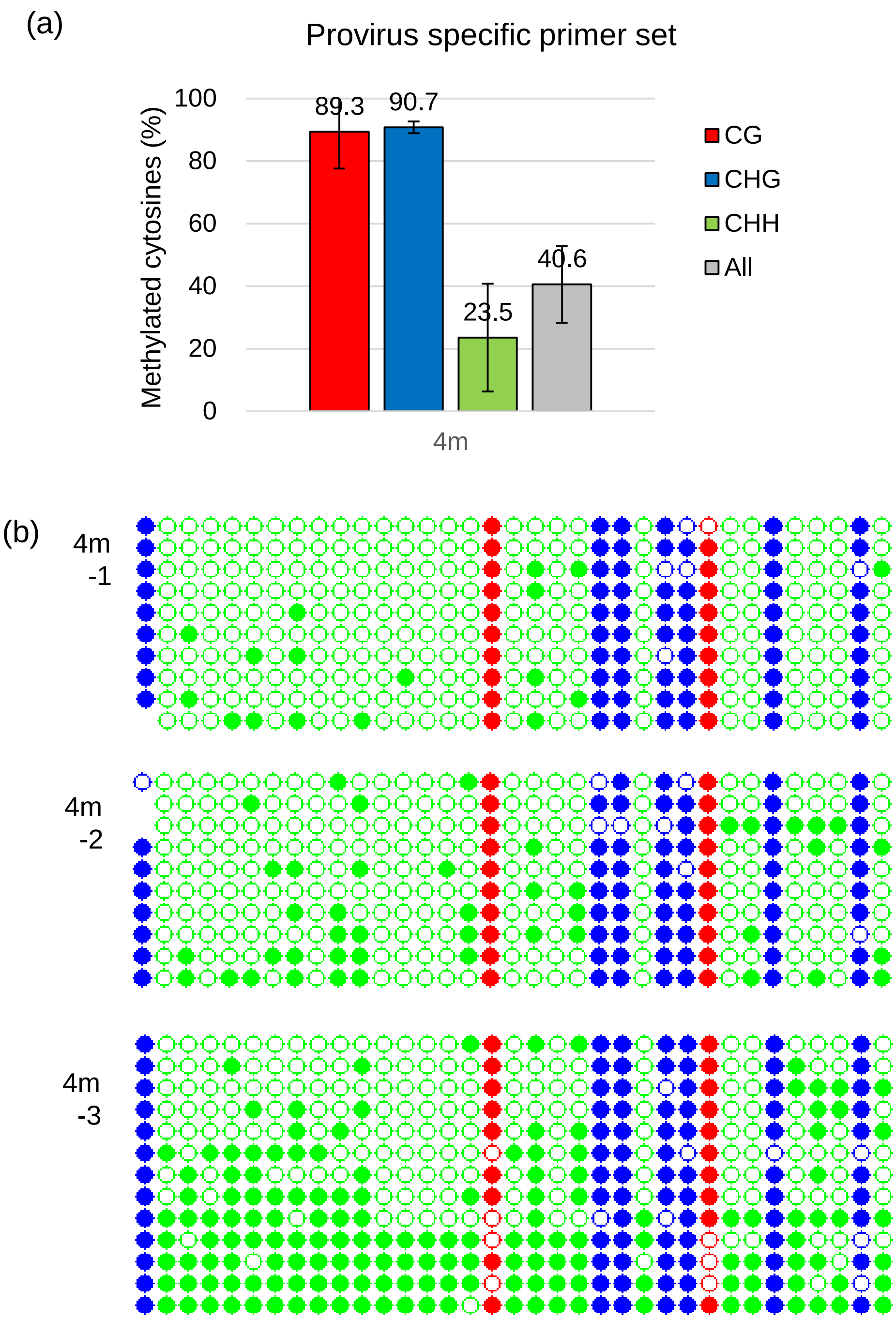

Figure S6. Methylation states of the promoter region of proviral PVCV analyzed using the bisulfite sequencing method. Genomic DNAs were prepared from leaves of three independent 4-month-old (4m) plants and treated using a bisulfite reagent. The promoter region of proviral PVCV was amplified by PCR with provirus-specific primers (Fig. S1 and Table S3), and sequenced. (a) The ratios of methylated cytosines in CG (red), CHG (blue) and CHH (green) sites were calculated from sequencing data of approximately 30 PCR clones. (b) Detailed presentation of methylated cytosines that are indicated as colored circles. Filled color circles, red (CG), blue (CHG), and green (CHH), indicate methylated cytosines, and open circles indicate unmethylated cytosines.

Figure S7

Episomal PVCV DNA in leaves

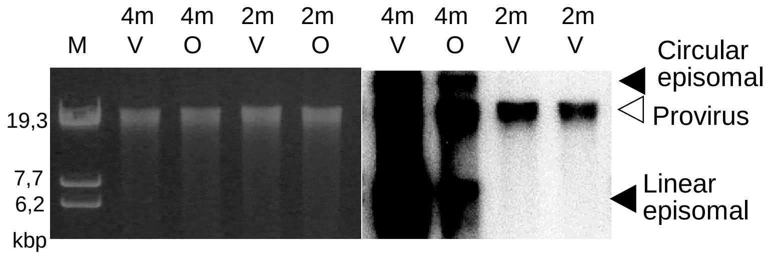

Figure S7. Detection of episomal PVCV DNA in leaves. DNA was isolated from the mid-vein and its surrounding regions (2m V) and the other regions (2m O) of leaves of 2-month-old plants and the mid-vein and its surrounding regions (4m V) and the other regions (4m O) of leaves with vein-clearing symptoms of 4-month-old plants. Proviral (a white arrowhead) and episomal (black arrowheads) PVCV DNAs were detected by Southern blot hybridization.

Fig. S8

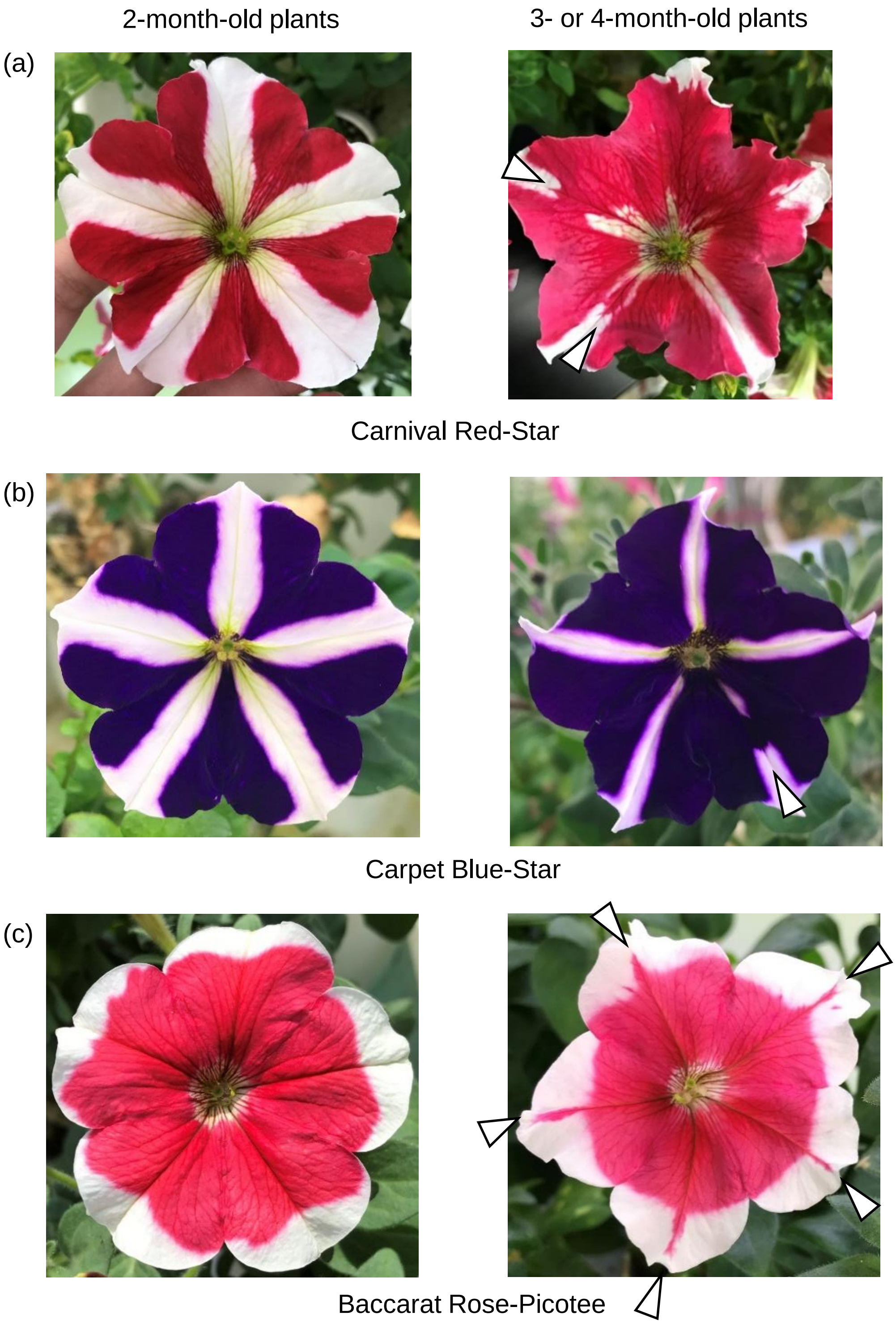

Figure S8. Occurrence of blotched flowers as the host plant ages. Blotched flowers were observed in petunia plants of three cultivars (a) Carnival Red-Star, (b) Carpet Blue-Star, and (c) Baccarat Rose-Picotee during long-term cultivation.
