# Supplementary material for: Disturbance of floral colour pattern by activation of an endogenous pararetrovirus, petunia vein clearing virus, in aged petunia plants
Source: Plant J. 2020 Apr 3;103(2):497–511. doi: 10.1111/tpj.14728 (PMC7496347; doi:10.1111/tpj.14728)
Supplement: Supplementary file 2 — Table S1. Ratios of PVCV reads per total reads in RNA‐seq datasets. Table S2. Ratios of proviral and episomal PVCV sequences in RNA‐seq datasets prepared from three independent 4‐month‐old plants of petunia cv Rondo Rose‐Star. Table S3. Sequences of oligo DNAs. [file TPJ-103-497-s002.docx]

Table S1. The ratios of PVCV reads per total reads in RNA-seq datasets

|  | 2-month-old plants | | | 4-month-old plants | | |
| --- | --- | --- | --- | --- | --- | --- |
| plant | PVCV | total | ratio | PVCV | total | ratio |
| No. 1 | 2355 | 13523523 | 1.74E-04 | 43567 | 12934381 | 0.00337 |
| No. 2 | 26 | 12840093 | 2.02E-06 | 21398 | 10658169 | 0.00201 |
| No. 3 | 904 | 13071619 | 6.92E-05 | 171731 | 12499446 | 0.0137 |

The ratios of PVCV reads per total reads in six RNA-seq datasets that were obtained from total RNAs purified from three 2-month-old and three 4-month-old plants of petunia cv. Rondo Rose-Star.

Table S2. The ratios of proviral and episomal PVCV sequences in RNA-seq datasets prepared from three independent 4-month-old plants of petunia cv. Rondo Rose-Star

| 4m-1 | **Nucleotides** | | **Number of reads** | | | **Ratio** | | |
| --- | --- | --- | --- | --- | --- | --- | --- | --- |
|  | Provirus | Episomal | Pro-reads | Epi-reads | other-  reads | Provirus | Episomal | other |
| 1 | T | A | 245 | 358 | 1 | 0.41 | 0.59 | 0.00 |
| 2 | C | T | 156 | 71 | 0 | 0.69 | 0.31 | 0.00 |
| 3 | C | T | 180 | 71 | 0 | 0.72 | 0.28 | 0.00 |
| 4 | A | G | 137 | 108 | 1 | 0.56 | 0.44 | 0.00 |
|  | G | A | 147 | 74 | 0 | 0.67 | 0.33 | 0.00 |
|  | G | T | 125 | 62 | 3 | 0.66 | 0.33 | 0.02 |
| 5 | - | C | 16 | 4 | 1 | 0.76 | 0.19 | 0.05 |
|  | - | T | 16 | 4 | 1 | 0.76 | 0.19 | 0.05 |
| 6 | G | A | 31 | 10 | 0 | 0.76 | 0.24 | 0.00 |
| 7 | A | G | 12 | 18 | 0 | 0.40 | 0.60 | 0.00 |
| 8 | - | A | 15 | 21 | 0 | 0.42 | 0.58 | 0.00 |
| 9 | G | T | 18 | 44 | 0 | 0.29 | 0.71 | 0.00 |
| average |  |  |  |  |  | 0.59 | 0.40 | 0.01 |

| 4m-2 | **Nucleotides** | | **Number of reads** | | | **Ratio** | | |
| --- | --- | --- | --- | --- | --- | --- | --- | --- |
|  | Provirus | Episomal | Pro-reads | Epi-reads | other-  reads | Provirus | Episomal | other |
| 1 | T | A | 540 | 15 | 1 | 0.97 | 0.03 | 0.00 |
| 2 | C | T | 380 | 5 | 0 | 0.99 | 0.01 | 0.00 |
| 3 | C | T | 396 | 4 | 0 | 0.99 | 0.01 | 0.00 |
| 4 | A | G | 377 | 10 | 3 | 0.97 | 0.03 | 0.01 |
|  | G | A | 369 | 11 | 1 | 0.97 | 0.03 | 0.00 |
|  | G | T | 253 | 2 | 5 | 0.97 | 0.01 | 0.02 |
| 5 | - | C | 36 | 2 | 1 | 0.92 | 0.05 | 0.03 |
|  | - | T | 36 | 2 | 1 | 0.92 | 0.05 | 0.03 |
| 6 | G | A | 89 | 1 | 0 | 0.99 | 0.01 | 0.00 |
| 7 | A | G | 29 | 1 | 0 | 0.97 | 0.03 | 0.00 |
| 8 | - | A | 34 | 1 | 1 | 0.94 | 0.03 | 0.03 |
| 9 | G | T | 29 | 3 | 0 | 0.91 | 0.09 | 0.00 |
| average |  |  |  |  |  | 0.96 | 0.03 | 0.01 |

| 4m-3 | **Nucleotides** | | **Number of reads** | | | **Ratio** | | |
| --- | --- | --- | --- | --- | --- | --- | --- | --- |
|  | Provirus | Episomal | Pro-reads | Epi-reads | other-  reads | Provirus | Episomal | other |
| 1 | T | A | 2351 | 6960 | 1 | 0.25 | 0.75 | 0.00 |
| 2 | C | T | 1267 | 1610 | 5 | 0.44 | 0.56 | 0.00 |
| 3 | C | T | 1543 | 1602 | 4 | 0.49 | 0.51 | 0.00 |
| 4 | A | G | 831 | 2155 | 16 | 0.28 | 0.72 | 0.01 |
|  | G | A | 922 | 1564 | 8 | 0.37 | 0.63 | 0.00 |
|  | G | T | 1116 | 1097 | 58 | 0.49 | 0.48 | 0.03 |
| 5 | - | C | 188 | 487 | 39 | 0.26 | 0.68 | 0.05 |
|  | - | T | 188 | 483 | 41 | 0.26 | 0.68 | 0.06 |
| 6 | G | A | 571 | 1107 | 5 | 0.34 | 0.66 | 0.00 |
| 7 | A | G | 239 | 1385 | 8 | 0.15 | 0.85 | 0.00 |
| 8 | - | A | 231 | 1295 | 12 | 0.15 | 0.84 | 0.01 |
| 9 | G | T | 247 | 1750 | 2 | 0.12 | 0.88 | 0.00 |
| average |  |  |  |  |  | 0.30 | 0.69 | 0.01 |

The ratios were calculated from number of reads on nine distinguishable sites between proviral and episomal sequences, which are shown in Figure S1, in RNA-seq datasets.

Table S3. Sequences of oligo DNAs

| Oligonucleotide | Sequence (5′-3′) |
| --- | --- |
| **qPCR** | |
| 26S rRNA F | AGCTCGTTTGATTCTGATTTCCAG |
| 26S rRNA R | GATAGGAAGAGCCGACATCGAAGG |
| Actin7 F | GACATTCAGCCCCTTGTTTG |
| Actin7 R | CGACCAACGATACTGGGAAA |
| PVCV F | CCCATTGCCTAAAATCCACA |
| PVCV R | CGTTCCAAAGTGCCTGAAAT |
| CHS-A F | TTCAGCAGCCCAAACTCTTC |
| CHS-A R | CAATCAGAAATGCCCAAAGG |
| Actin2 (*N. benthamiana*) F | CTTGAAACAGCAAAGACCAGC |
| Actin2 (*N. benthamiana*) R | GGAATCTCTCAGCACCAATGG |
| GFP F | AACTTCAAGATCCGCCACAA |
| GFP R | CTCGTCCATGCCGTGAGT |
| **Southern / Northern hybridization** | |
| PVCV ORF F | GTATCAGAGCCATCCATTTTGTTGA |
| PVCV ORF R | CATGCTTTAGACACATAGGACCACTACT |
| PVCV promoter F | AGTTTGTCTAGTCTACTTCTGTCT |
| PVCV promoter R | AGATTGAACCTATAAGAGAATTCTGA |
| CHS-A F | GTGGTTGAAGTGCCCAAAC |
| CHS-A R | GCCCAAATCCAAAAAGAACA |
| **Cloning of PVCV sequence** | |
| PVCV promoter F | AGTTTGTCTAGTCTACTTCTGTCT |
| PVCV promoter R | AGATTGAACCTATAAGAGAATTCTGA |
| **Bisulfite sequencing** | |
| Positive control cloning F | TGTGCCTGCATTGAGAGTG |
| Positive control cloning R | CACCCAAGGTTCTTATGTTTCA |
| Provirus selective PVCV F | GGTAAYYTYAYTAGTTGTTTTYAGG |
| Provirus selective PVCV R | CTATAARARAATTCTRATTAAACTRARRC |
| Non selective PVCV F | GAGTYTGTATTATTAAGTGTGATAAGTG |
| Non selective PVCV R | CCTACTATCTTCARTAATCACTCTTATA |
| CHS-A promoter F | TAGAAGTGAYAGAAATYATATGTAAGAATG |
| CHS-A promoter R | ATACTCCTCTACARTCACCATTTTT |
| Sequencing primer (U-19) | GTTTTCCCAGTCACGACGT |
| **Cloning for agroinfiltration** | |
| PVCV ORF inf F | CACTGTTGATACATATGACTTCTCCTTCTGATTATCAATCC |
| PVCV ORF inf R | ATTCAGAATTGTCGACTTAGACACATGGTTCTGATGAGCTGT |
| PVCV C’ inf F | CACTGTTGATACATATGTTTCAAAGGAGGACAAGATC |
| PVCV C’ inf R | ATTCAGAATTGTCGACTTAGACACATGGTTCTGATGAGCTGT |
